# Supplementary material for: Surgical informed consent practice and associated factors among adult postoperative patients in public hospitals of Mekelle, Tigray, Ethiopia 2023/2024. Cross sectional
Source: PLoS One. 2025 Feb 3;20(2):e0310920. doi: 10.1371/journal.pone.0310920 (PMC11790128; doi:10.1371/journal.pone.0310920)
Supplement: Attachment — Submitted filename: PONE-D-24-22632_R1&R2.pdf [file pone.0310920.s004.pdf]

# PLOS ONE

## Surgical informed consent practice and associated factors among adult postoperative patients in public hospitals of Mekelle, North Ethiopia 2023/2024. Cross sectional

--Manuscript Draft--

| | |
|---|---|
| **Manuscript Number:** | PONE-D-24-22632R1 |
| **Article Type:** | Research Article |
| **Full Title:** | Surgical informed consent practice and associated factors among adult postoperative patients in public hospitals of Mekelle, North Ethiopia 2023/2024. Cross sectional |
| **Short Title:** | Surgical informed consent practice and factors among postoperative in public hospitals of Mekelle,Ethiopia. |
| **Corresponding Author:** | fiseha Abadi Gebreanenia, Msc<br>Aksum University College of Health Science and Medicine<br>Aksum, Tigray ETHIOPIA |
| **Keywords:** | Practice, Surgical informed consent, Patient |
| **Abstract:** | Background<br>Substantial weaknesses and omissions of surgical informed consent are evident and the current elements of the surgical informed consent process are largely neglected in daily practice. This study aimed to assess surgical informed consent practice and associated factors among adult postoperative patients in public hospitals of Mekelle, Ethiopia.<br>Methods<br>Institution based cross-sectional study was conducted among 314 adult postoperative patients in public hospitals of Mekelle, Ethiopia. Descriptive, bivariate and multivariable logistic regression analyses were performed using statistical package for social science version 27. The adjusted odds ratio with a 95% confidence interval and p-value <0.05 was used to declare significant association.<br>Result<br>Only 35.8% (CI 95%, 30.6, 41) of the respondents were identified to have received the recommended (6 or more) components of surgical informed consent. Educational level [AOR 5.76 (1.02, 32.6)], time of surgical informed consent delivery [AOR 3.27 (1.5, 7.11)], qualification of counselor who took surgical informed consent them [AOR3.185 (1.21, 8.38)], hospital type [AOR 2.85 (1.26, 6.46)], and duration of counseling [AOR 6.9 (3.33, 14.3)] were statistically significant factors.<br>Conclusion<br>Majority of participants did not receive comprehensive information during the surgical informed consent process in the study hospitals. To improve the delivery it is suggested that health professionals; create rapport with patient, spend more time during counseling, and deliver it early. |
| **Order of Authors:** | Fiseha Abadi Gebreanenia, Msc |
| | Hailemarim Berhe Kahsay, phD |
| | Desta siyoum Belay, Msc, Assistant professor |
| | Binyam Gebrehiwet Tesfay, Msc |
| | Fissha Brhane Mesele, Msc |
| | Mamush gidey Abirha, Msc |
| **Response to Reviewers:** | To; Kahsu Gebrekidan, Ph.D.<br>Academic Editor<br>PLOS ONE |
| **Additional Information:** | |
| **Question** | **Response** |
| **Financial Disclosure** | The author(s) received no specific funding for this work. |

*Powered by Editorial Manager® and ProduXion Manager® from Aries Systems Corporation*

| | |
|---|---|
| Enter a financial disclosure statement that describes the sources of funding for the work included in this submission. Review the submission guidelines for detailed requirements. View published research articles from *PLOS ONE* for specific examples.<br><br>This statement is required for submission and **will appear in the published article** if the submission is accepted. Please make sure it is accurate.<br><br>**Funded studies**<br>Enter a statement with the following details:<br>• Initials of the authors who received each award<br>• Grant numbers awarded to each author<br>• The full name of each funder<br>• URL of each funder website<br>• Did the sponsors or funders play any role in the study design, data collection and analysis, decision to publish, or preparation of the manuscript?<br><br>Did you receive funding for this work? | |
| **Competing Interests**<br><br>Use the instructions below to enter a competing interest statement for this submission. On behalf of all authors, disclose any competing interests that could be perceived to bias this work—acknowledging all financial support and any other relevant financial or non-financial competing interests.<br><br>This statement is required for submission and **will appear in the published article** if the submission is accepted. Please make sure it is accurate and that any funding sources listed in your Funding Information later in the submission form are also declared in your Financial Disclosure statement.<br><br>View published research articles from | This journal is very preferable because of its quality paper publishing especially in health-related researches. |

| | |
|---|---|
| *PLOS ONE* for specific examples.<br><br>**NO authors have competing interests**<br><br>Enter: *The authors have declared that no competing interests exist.*<br><br>**Authors with competing interests**<br><br>Enter competing interest details beginning with this statement:<br><br>*I have read the journal's policy and the authors of this manuscript have the following competing interests: [insert competing interests here]*<br><br>* typeset | |
| **Ethics Statement**<br><br>Enter an ethics statement for this submission. This statement is required if the study involved:<br><br>• Human participants<br>• Human specimens or tissue<br>• Vertebrate animals or cephalopods<br>• Vertebrate embryos or tissues<br>• Field research<br><br>Write "N/A" if the submission does not require an ethics statement.<br><br>General guidance is provided below. Consult the submission guidelines for detailed instructions. **Make sure that all information entered here is included in the Methods section of the manuscript.** | ethical clearance was obtained from institutional review board of Mekelle University. with approval number MU-IRB 2123/2023, and written consent was obtained from study participants. |

Powered by Editorial Manager® and ProduXion Manager® from Aries Systems Corporation

| | |
|---|---|
| **Format for specific study types**<br><br>**Human Subject Research (involving human participants and/or tissue)**<br>• Give the name of the institutional review board or ethics committee that approved the study<br>• Include the approval number and/or a statement indicating approval of this research<br>• Indicate the form of consent obtained (written/oral) or the reason that consent was not obtained (e.g. the data were analyzed anonymously)<br><br>**Animal Research (involving vertebrate animals, embryos or tissues)**<br>• Provide the name of the Institutional Animal Care and Use Committee (IACUC) or other relevant ethics board that reviewed the study protocol, and indicate whether they approved this research or granted a formal waiver of ethical approval<br>• Include an approval number if one was obtained<br>• If the study involved *non-human primates*, add *additional details* about animal welfare and steps taken to ameliorate suffering<br>• If anesthesia, euthanasia, or any kind of animal sacrifice is part of the study, include briefly which substances and/or methods were applied<br><br>**Field Research**<br><br>Include the following details if this study involves the collection of plant, animal, or other materials from a natural setting:<br>• Field permit number<br>• Name of the institution or relevant body that granted permission | |
| **Data Availability**<br><br>Authors are required to make all data underlying the findings described fully available, without restriction, and from the time of publication. PLOS allows rare exceptions to address legal and ethical concerns. See the PLOS Data Policy and FAQ for detailed information. | Yes - all data are fully available without restriction |

| | |
|---|---|
| A Data Availability Statement describing where the data can be found is required at submission. Your answers to this question constitute the Data Availability Statement and **will be published in the article**, if accepted.<br><br>**Important:** Stating 'data available on request from the author' is not sufficient. If your data are only available upon request, select 'No' for the first question and explain your exceptional situation in the text box.<br><br>Do the authors confirm that all data underlying the findings described in their manuscript are fully available without restriction? | |
| **Describe where the data may be found in full sentences. If you are copying our sample text, replace any instances of XXX with the appropriate details.**<br><br>• If the data are **held or will be held in a public repository**, include URLs, accession numbers or DOIs. If this information will only be available after acceptance, indicate this by ticking the box below. For example: *All XXX files are available from the XXX database (accession number(s) XXX, XXX.*).<br>• If the data are all contained **within the manuscript and/or Supporting Information files**, enter the following: *All relevant data are within the manuscript and its Supporting Information files.*<br>• If neither of these applies but you are able to provide **details of access elsewhere**, with or without limitations, please do so. For example:<br><br>*Data cannot be shared publicly because of [XXX]. Data are available from the XXX Institutional Data Access / Ethics Committee (contact via XXX) for researchers who meet the criteria for access to confidential data.*<br><br>*The data underlying the results presented in the study are available from (include the name of the third party* | All relevant data are within the manuscript and its Supporting Information files. |

Powered by Editorial Manager® and ProduXion Manager® from Aries Systems Corporation

| | |
|---|---|
| *and contact information or URL).*<br>• This text is appropriate if the data are owned by a third party and authors do not have permission to share the data.<br><br>* typeset | |
| Additional data availability information: | Tick here if the URLs/accession numbers/DOIs will be available only after acceptance of the manuscript for publication so that we can ensure their inclusion before publication. |

Powered by Editorial Manager® and ProduXion Manager® from Aries Systems Corporation

Manuscript Click here to access/download;Manuscript;Munscript.docx

**Research article**

# Surgical informed consent practice and associated factors among adult postoperative patients in public hospitals of Mekelle, Tigray, Ethiopia 2023/2024. Cross sectional

**Authors**

Fiseha Abadi Gebreanenia[1*, #a], Hailemarim Berhe Kahsay[2, #b], Desta Siyoum Belay[2, #b], Binyam Gebrehiwet Tesfay[4, #c], Fissha Brhane Mesele[3,#b] , Mamush Gidey Abrha[4,#c]

[1] Aksum University, college of health science, department of nursing, Axum, Ethiopia

[2] Mekelle University, College of health science, School of nursing, Mekelle, Ethiopia

[3] Gambella teachers' education and health Science College

[4] Adigrat University, college of medicine and health science, Department of nursing, Adigrat, Ethiopia

[#a] current address: Aksum, Northern Ethiopia

[#b] current address: Mekelle, Northern Ethiopia

[#c] current address: Adigrat, Northern Ethiopia

*Fiseha@aku.edu.et (FA)

# Abstract

**Background**

Substantial weaknesses and omissions of surgical informed consent are evident and the current elements of the surgical informed consent process are largely neglected in daily practice. This study aimed to assess surgical informed consent practice and associated factors among adult postoperative patients in public hospitals of Mekelle, Tigray, Ethiopia.

**Methods**

Institution based cross-sectional study was conducted among 314 adult postoperative patients in public hospitals of Mekelle, Tigray, Ethiopia. Descriptive, bivariate and multivariable logistic regression analyses were performed using statistical package for social science version 27.

**Result**

Only 35.8% (CI 95%, 30.6, 41) of the respondents were identified to have received the recommended (6 or more) components of surgical informed consent. Educational level [AOR 5.76 (1.02, 32.6)], timing of surgical informed consent delivery [AOR 3.27 (1.5, 7.11)], qualification of counselor who took surgical informed consent them [AOR3.185 (1.21, 8.38)], hospital type [AOR 2.85 (1.26, 6.46)], and duration of counseling [AOR 6.9 (3.33, 14.3)] were statistically significant factors.

**Conclusion**

Majority of participants did not receive comprehensive information during the surgical informed consent process in the study hospitals. To improve the delivery it is suggested that health professionals; create rapport with patient, spend more time during counseling, and deliver it early.

# Introduction

Informed consent is a process that includes providing the patient with information about his or her health status, diagnosis, treatment plans, treatment options, and prognosis to make voluntary and informed decision about his/her treatment(1). As part of Informed consent, surgical informed consent (SIC) is the process whereby patients are informed of all the necessary information about the general health, type of surgery, subsequent treatment plans, risk and alternatives. Informed consent is built by competent patient to make decision about one's surgical procedure with enough information which is clear, understandable and valid((2, 3).

Surgical informed consent is improperly practiced and violated in many occasions; this is mostly due to lack of health care provider awareness and experience with SIC, heavy workload and negligence of health care providers, and lack of a standard consent form (4, 5). While evidence suggests that every surgical patient should be informed by the performing healthcare provider about the proposed surgery before signing a consent form, this isn't always the case in clinical settings, particularly in developing countries (6).

A study in Italy revealed that 84.5% of the participants either personally or through a delegate signed the informed consent. Most attention was given to the diagnosis and the type of surgical procedure, which was communicated respectively to 92.8 and 88.2% of the patients (10).

Another study conducted in Nigeria among surgeons revealed that, 54.9% of the surgeons agreed that sufficient information is not provided to patients while obtaining their consent for surgical procedures (7).

In Ethiopia a cross-sectional study conducted on Surgical informed consent using 13 components of SIC showed that, significant proportion (73.5%) of the respondents hasn't received at least six

of the 13 components of SIC suggested by the investigators which is unacceptable (12). Similar study conducted in Addis Ababa among patients undergone caesarian section 44.9% received the minimum required components of surgical informed consent (8, 9). This could be severe in war torn Tigray as Only 9.7% of health centers, 43.8% of general hospitals and 21.7% of primary hospitals are functional and many health professionals fled out of the region (10).

If adequate information is not provided, the legal consequences could potentially involve lawsuit, professional licensure actions, and negative accreditation implications for the provider and the institution wherein the treatment occurred (1).

According to previous studies factors like educational level, type of surgery, time of counseling, schedule of surgery and duration of counseling were significantly associated with SIC practice (4, 8, 9, 11).

To alleviate the problem the ministry of health of Ethiopia has launched and formulated strategies for healthcare ethics education for students and healthcare professional's development, this program is called 'Motivated, Competent and Compassionate Health Workforce' (12). However, few health care professionals were trained and its applicability on the ground has been poor (13).

While several studies in Ethiopia have examined patients' knowledge and perception of surgical informed consent, few have investigated the actual practice of informed consent as experienced by patients. Additionally, existing research has primarily focused on specific specialties and among healthcare professionals. In addition to this there is no study conducted in the study area and in the region as well. This study aims to address this gap by investigating surgical informed consent practice and its associated factors from patient perspective among adult surgical patients in

Mekelle public hospitals. This study aimed to address the gap in research by determining the prevalence and associated factors of workplace violence against nurses in Mekelle, Ethiopia.

This study's findings will benefit participants by raising their awareness of legal proceedings regarding their treatment. Healthcare providers will gain insight into their current SIC practices and can use this information to improve their services, ultimately minimizing litigation and medico-legal issues. Regulators and ethical committees can utilize this study's findings to assess current SIC practices and identify potential gaps. Additionally, the research can serve as valuable baseline data for future studies with similar interests.

# Methods and materials

## Study design, population, area and period

An institution-based cross-sectional study design was employed for this study. The study was conducted in public hospitals located in Mekelle, the capital city of Tigray Regional State, Ethiopia. The average number of major surgeries performed monthly in Mekelle is approximately 1001. Data was collected from December 20, 2023, to January 20, 2024, G.C. The study population included all adult postoperative patients admitted to public hospitals in Mekelle during the data collection period.

## Eligibility criteria

### Inclusion criteria

- All admitted adult postoperative patients were included in the study.

### Exclusion criteria

- The study excluded patients who were not fully conscious during data collection or whose relatives who signed the consent were unavailable.
- Patients who had undergone repeated surgery were excluded to avoid confounding between the two informed consent processes.

## Sample size determination

The actual sample size for the study was determined using single population proportion formula taking 26.5%(patients who received at least six components of SIC) proportion  taken from a research conducted in Hawassa (8); with 95% confidence interval (1.96); α=0.05 ;5% marginal of error (5%).

$$n = \frac{(Z\alpha/2)2 * p\ (1-p)}{d^2}$$

Where:-

n= required sample size

z= critical value at 95% CI (1.96)

p= prevalence rate, 26.5% i.e. 0.265

Margin of error (d) to be 5% (d = 0.05)

$$n = \frac{(1.96)^2 * 0.265(1-0.265)}{(0.05)^2} \quad n = 299$$

By adding 5% (15) of non-response rate the final sample size was **314.**

## Sampling procedure and technique

All public hospitals in Mekelle city offering surgical services were selected for the study. Within each hospital, the sample size for each surgical specialty was proportionally allocated based on the average monthly number of major surgeries performed (N). Participants were chosen using systematic random sampling with a predetermined sampling interval (K) which is three. The K value was determined by dividing the number of average surgeries done in all public hospital to the sample size. In selected hospitals and wards, participants were interviewed based on their sequence of operations done for each ward every K intervals. *(figure1)*

## Data collection method and tools

Data was collected by four nursing students and one graduated BSc nurse supervisor with no affiliation to the study hospitals, and it was collected using an interviewer administered

questionnaire. The questionnaire was developed from various sources (2, 8, 9, 14). It included three main sections: socio-demographic characteristics, service-related characteristics, and thirteen essential components of SIC based on recommendation from 'Royal College of Surgeons standards of informed consent practices for surgical procedures'. Data collection spanned one month, with interviews and document reviews conducted daily on working days, starting from the first postoperative day and continued until patient discharge.

# Variables

# Dependent variables

- Surgical informed consent practice

## Independent variable

- Socio demographic characteristic (age, sex, marital status, educational level etc...
- Service related characteristics
    - schedule of surgery (emergency/elective),
    - Type of hospital(general, specialized)
    - Timing of SIC delivery (immediately before surgery, one day before surgery...)
    - Type of surgery(surgical, gyn/obs, orthopedic)
    - Duration of counseling( <5minutes, >5minutes)
    - Qualification of health professionals (senior physician, resident/GP, Nurse...)

# Operational definitions

**Practice of SIC:** is defined as receipt of six or more (the minimum recommendation) components from the total thirteen components of the SIC (8, 9).

- **Thirteen components of SIC**

1. Respondent/respondent’s family was requested for surgical informed consent.
2. Respondent/respondent’s family signed on a surgical informed consent form.
3. Respondent was informed why the surgery will be performed (indication of surgery).
4. Respondent was informed the expected time the surgery will take.
5. Respondent was informed about presence/absence of alternative treatment options.
6. Respondent was informed about type of anesthesia to be used.
7. Respondent was given counseling aids which assist in decision making.
8. Respondent was informed about potential complication/s which may arise.
9. Respondent was informed about consequences of refusing the proposed surgery.
10. There was a favorable environment to say “No” to the proposed surgery.
11. Respondent was given adequate time for decision to sign on the informed consent form.
12. Respondent given opportunity to choose from anesthesia options.
13. Respondent was given an opportunity to ask question.

## Data analysis and management

The data was coded and entered in to Epi Info version 7.2. After cross-checking for consistency and accuracy, the data was cleaned and exported to SPSS version 27 for statistical analysis. Descriptive statistics were performed, Frequency distributions and percentages were employed for categorical variables and the results were summarized using tables, text, and figures.

Both bivariable and multivariable logistic regression analyses were used to determine the association between independent variable and dependent variable. In Bivariable analysis variables having p value less than 0.2 were entered in to multivariable logistic regression to assess statistical

association between the outcome variable and independent variables. In multivariable logistic regression, p value <0.05 with 95% confidence interval (CI) were considered statistically significant. The Hosmer- Lemeshow goodness-of-fit model coefficients tests procedure was used to test for model fitting. The assumptions of logistic regression was checked before proceeding to regression.

## Data quality assurance

To ensure data quality, a standard questionnaire relevant to this study was obtained from published journal articles. The English questionnaire was translated into Tigrigna, and the Tigrigna version was then back-translated into English to maintain consistency. However, to meet the objectives of the study, Pretest were done in 5% of sample size in Axum Referral Hospital and Kidiste Mariyam Hospital one week before scheduled data collection day to improve the tool. To standardize data collection techniques, the data collectors received a one-day training session covering the questionnaire's principles, ethical considerations, and question meanings. The supervisor and principal investigator closely supervised the data collectors during data collection. Each completed questionnaire was immediately checked for errors, completeness and legibility.

## Ethical considerations

Official ethical clearance letter was obtained from the institutional Review board of the College of Health Sciences of the Mekelle University, after approval the necessary communication were made with chief clinical directors, medical directors and the hospital administrators of each hospital. Written informed consent were obtained from the respective personnel before starting study. Filled questionnaires kept securely and only accessible to the researcher. All methods were performed in accordance with declarations of Helsinki.

# Result

In this study, a total of 310 out of 314 samples were included, yielding a response rate of 98.7%.

## Socio demographic characteristics of study participants

Out of the 310 post-operative patients interviewed, 160 (51.6%) were males. From the total respondents 140 (45.2%) were between the ages of 26 and 40 years. Out of the total study participants (87.4%) were Orthodox followers and (72.3%) of them were married. *(Table 1)*

**Table 1: Socio-demographic characteristics of respondents to assess SIC practice among adult postoperative patients in public hospitals of Mekelle, Ethiopia 2023/2024 (n=310)**

| Variable | Category | Frequency | % |
|---|---|---|---|
| Sex | Male | 160 | 51.6 |
| | Female | 150 | 48.4 |
| | Total | 310 | 100 |
| Religion | Orthodox | 271 | 87.4 |
| | Muslim | 36 | 11.6 |
| | Catholic | 2 | 0.6 |
| | Protestant | 1 | 0.3 |
| | Total | 310 | 100 |
| Marital status | Married | 224 | 72.3 |
| | Single | 49 | 15.8 |
| | Divorced | 19 | 6.1 |
| | Widowed | 15 | 4.8 |
| | Total | 310 | 100 |
| Educational level | Unable to read and write | 92 | 29.7 |
| | Able to read and write | 47 | 15.2 |
| | Primary(1-8) | 54 | 17.4 |
| | Secondary (9-12) | 64 | 20.6 |
| | Diploma | 27 | 8.7 |
| | Degree | 23 | 7.4 |
| | Master and above | 3 | 1 |
| | Total | 310 | 100 |

| | | | |
|---|---|---|---|
| Occupation | Government employee | 45 | 14.5 |
| | Private employee | 18 | 5.8 |
| | Farmer | 114 | 36.8 |
| | Housewife | 72 | 23.2 |
| | Private business | 23 | 7.4 |
| | Daily laborer | 13 | 4.2 |
| | Other | 25 | 8.1 |
| | Total | 310 | 100 |
| Residence | Urban | 146 | 47.1 |
| | Rural | 164 | 52.9 |
| | Total | 310 | 100 |

## Service related characteristics of the respondents

Nearly half of the patients (49.4%) who underwent surgery were unaware of qualifications of the healthcare provider who counseled them about surgical informed consent (SIC). The counseling session duration for most patients (77.7%) was less than 5 minutes. Over half of the surgeries were emergency procedures (59.4%). Out of the total surgeries done (43.5%) were general surgery. Additionally, out of the total surgeries in this study 194(62.6%) were performed at Ayder Specialized Hospital. *Table 2*

**Table 2: Service related characteristics of the respondents to assess SIC practice among adult postoperative patients in public hospitals of Mekelle, Ethiopia 2023/2024 (n=310)**

| Variable | Category | Frequency | Percent |
|---|---|---|---|
| Duration of counseling | <05 minutes | 241 | 77.7 |
| | >05 minutes | 69 | 22.3 |
| | Total | 310 | 100 |
| Qualification of counselor who took surgical informed consent | Senior physician | 68 | 2 |
| | Resident physician | 29 | 9.4 |
| | Intern or GP | 5 | 1.6 |
| | Nurse | 32 | 10.3 |
| | Midwife | 23 | 7.4 |
| | Did not know | 153 | 49.4 |
| | Total | 310 | 100 |
| Schedule of surgery | Elective | 126 | 40.6 |
| | Emergency | 184 | 59.4 |
| | Total | 310 | 100 |
| Type of hospital | Specialized | 194 | 62.6 |
| | General | 116 | 37.4 |
| | Total | 310 | 100 |
| Type of anesthesia | General | 137 | 44.2 |
| | Spinal | 173 | 55.8 |
| | Total | 310 | 100 |
| Timing of counseling for informed consent | The day before date of surgery | 109 | 35.2 |
| | On the day of surgery | 58 | 18.7 |
| | Immediately before surgery | 126 | 40.6 |
| | On the operation table | 17 | 5.5 |
| | Total | 310 | 100 |
| Type of surgery | General surgery | 135 | 43.5 |
| | Orthopedic | 64 | 20.6 |
| | Gynecology/obstetric | 111 | 35.9 |
| | Total | 310 | 100 |

## Components of surgical informed consent received by respondents

Almost all (99%) of the respondents or their family members signed an informed consent form. Most respondents (87.7%) were informed about the indication for the surgery. However, only 8.4% of respondents were informed about the expected surgery duration, and very few respondents (1%) were given a chance to choose their anesthesia. More than half (55.5%) of respondents received information about potential complications during the procedure. *Table 3*

**Table 3: Essential components of surgical informed consent received by respondents to assess SIC practice among adult postoperative patients in public hospitals of Mekelle, Ethiopia 2023/2024 (n=310)**

| **Variables** | **Responses , n, %** | | |
|---|---|---|---|
| | Yes | No | Do not remember |
| Respondent/respondent’s family was requested for surgical informed consent | 303(97.7) | 5(1.6) | 2(0.6) |
| Respondent/respondent’s family signed on an surgical informed consent form | 307(99) | 3(1) | _ |
| Respondent was informed why the surgery will be performed (indication of surgery) | 272(87.8) | 33(10.6) | 5(1.6) |
| Respondent was informed the expected time the surgery will take | 26(8.4) | 273(88.1) | 11(3.5) |
| Respondent was informed about presence/absence of alternative treatment options | 47(15.2) | 254(81.9) | 9(2.9) |
| Respondent was informed about type of anesthesia to be used | 19(6.1) | 275(88.7) | 16(5.2) |
| Respondent was given counseling aids which assist in decision making | 140(45.2) | 163(52.6) | 7(2.2) |
| Respondent was informed about potential complication/s which may arise | 203(65.5) | 100(32.3) | 7(2.2) |
| Respondent was informed about consequences of refusing the proposed surgery | 179(57.7) | 125(40.3) | 6(2) |
| There was a favorable environment to say “No” to the proposed surgery | 58(18.7) | 248(80) | 4(1.3) |
| Respondent was given adequate time for decision to sign on the informed consent form | 164(52.9) | 139(44.8) | 7(2.3) |
| Respondent was given opportunity to choose from anesthesia options | 3(1) | 298(96.1) | 9(2.9) |
| Respondent was given an opportunity to ask question | 127(41) | 179(57.7) | 4(1.3) |

## Receipt of six or more components of surgical informed consent

Based on the total numbers of the components of SIC respondents received before their surgery, out of the total thirteen components 35.8% (CI =95%, 30.6, 41)of the respondents were identified to have received six or more (the minimum recommendation) components of surgical informed consent. *Figure 3*

## Factors associated with receipt of six or more components of surgical informed consent

In bi-variable regression analysis variables like educational level, occupation, sex, type of anesthesia, type of surgery, schedule of surgery, timing of SIC delivery, health care provider who took SIC, hospital type, and counselor time spent on informed consent were significant at P= <0.2, thus they were selected for multi-variable regression analysis. Following multi-variable regression analysis variables like educational level, timing of SIC delivery, Qualification of counselor who took SIC, hospital type, and counselor time spent on informed consent were significant at P<0.05. *Table 4*

**Table 4; Bi-variables and multi-variable analysis to assess factors associated with receipt of six or more components of SIC among adult postoperative patients in public hospitals of Mekelle, Ethiopia 2023/2024 n=310**

| Variable | Category | Received 6 or more components of SIC | | COR (95%CI) | AOR(95% CI) | P-value |
|---|---|---|---|---|---|---|
| | | Yes | No | | | |
| Educational level | able to read and write | 16 | 30 | 1.47(0.69,3.15) | 2.29(0.84,6.2) | 0.105 |
| | Primary(1-8) | 19 | 38 | 1.38(0.67,2.82 | 2.1(0.75,5.9) | 0.157 |
| | Secondary (9-12) | 19 | 41 | 1.28(0.63,2.64) | 2.5(0.68,8.2) | 0.129 |
| | Diploma | 15 | 13 | 3.18(1.33,7.62) | 5.1(0.93,28.15) | 0.06 |
| | Degree and above | 17 | 8 | 5.86(2.25,15.27) | **5.76(1.02,32.6)*** | **0.047*** |
| | Unable to read and write | 25 | 69 | Ref. | Ref. | |
| Duration of counseling | <05 minutes | 61 | 180 | Ref. | Ref. | |
| | >05 minutes | 50 | 19 | 7.765(4.25,14.19) | **65.9(3.33,14.3)*** | **<0.001*** |
| Qualification of counselor who took SIC | Did not know | 48 | 105 | Ref. | Ref. | |
| | Senior physician | 39 | 29 | 2.94(1.63,5.3) | **3.18(1.21,8.38)*** | **0.019*** |
| | Resident physician, Intern or GP | 10 | 24 | 0.91(0.4,2) | 0.445(0.15,1.35) | 0.153 |
| | Nurse | 9 | 23 | 0.856(0.37,1.99) | 1.89(0.59,6.1) | 0.287 |
| | Midwife | 5 | 18 | 0.6(0.21,1.73) | 1.34(0.3,6) | 0.704 |
| Type of hospital | General | 31 | 85 | Ref. | Ref. | |
| | Specialized | 80 | 114 | 1.92(1.16,3.17) | **2.85(1.26,6.46)*** | **0.012*** |
| Timing of counseling for informed consent | Immediately before surgery | 35 | 108 | Ref. | Ref. | |
| | The day before date of surgery | 59 | 50 | 3.64(2.13,6.22) | **3.27(1.5,7.11)*** | **0.003*** |
| | On the day of surgery | 17 | 41 | 1.28(0.65,2.53) | 0.86(0.36,2.1) | 0.751 |
| Occupation | Daily laborer | 4 | 9 | Ref. | Ref. | |
| | Private employee | 6 | 12 | 1.125(0.243,5.2) | 0.51(0.79,3.3) | 0.481 |
| | Farmer | 37 | 77 | 1.08(0.31,3.74) | 1.09(0.21,5.53) | 0.919 |
| | Housewife | 16 | 56 | 0.643(0.175,2.36) | 0.99(0.17,5.6) | 0.988 |
| | Private business | 5 | 18 | 0.625(0.134,2.9) | 0.57(0.85,3.84) | 0.564 |

| | | | | | | |
|---|---|---|---|---|---|---|
| | Government employee | 29 | 16 | 4.08(1.08,15.37) | 1.59(0.24,10.5) | 0.629 |
| | Other | 14 | 11 | 2.86(0.69,11.82) | 2.34(0.43,12.8) | 0.326 |
| Sex | Female | 48 | 102 | Ref. | Ref. | |
| | Male | 63 | 97 | 1.38(0.8,2.2) | 0.92(0.37,2.3) | 0.86 |
| Schedule of surgery | Elective | 51 | 75 | 1.4(0.88,2.25) | 1.01(0.49,2.07) | 0.977 |
| | Emergency | 60 | 124 | Ref. | Ref. | |
| Type of surgery | General surgery | 53 | 82 | Ref. | Ref. | |
| | Orthopedic | 30 | 34 | 1.37(0.75,2.5) | 1.28(0.55,2.98) | 0.565 |
| | Gynecology/obstetric | 28 | 83 | 0.52(0.3,0.9) | 0.76(0.24,2.4) | 0.641 |
| Type of anesthesia | General | 59 | 78 | 1.76(1.1,2.81) | 1.07(0.51,2.24) | 0.854 |
| | Spinal | 52 | 121 | Ref. | Ref. | |

***= Statistically significant with p- value < 0.2** **1=Reference group of the variable**

****=Statistically significant with p- value <0.05**

# Discussion

The receipt of six or more (the minimum recommendation) components of SIC in this study is (35.8%) with (CI 95%, 30.6, 41), which is slightly higher than the study conducted at Hawassa (26.5%) (8). This difference might be attributable to the study area covered, which exposes patients to various health professionals and different working environments. This is because this study included all public hospitals and post-op wards, whereas the latter study was exclusively conducted in the gynecology/obstetrics department of a specific hospital. On the other hand, it is lower than the study conducted in Addis Ababa (44.9%) (9). This disparity is likely due to decreased staff morale in helping patients in the study area, possibly stemming from a lack of incentives, service delivery arrangements, resource capacities and increased workload (5).

A multi-variable regression analysis identified several factors associated with receipt of six or more components of SIC. Participants with a degree or higher educational level were 5.7 times more likely to receive 6 or more components than those who were unable to read or write (AOR=5.76, 95%CI=1.02, 32.6, P=0.047)**.** These findings are in line with a study conducted in Iran, and Addis Ababa (AOR=2.4, 95%CI=1.81, 11.32) (9, 11). This difference might be attributable to the fact that educated participants tend to ask more questions. This, in turn, led them to receive more information about the proposed surgery.

Participants counseled for more than 5 minutes were almost seven times more likely to receive the 6 or more components of SIC than those counseled for less than 5 minutes (AOR= 6.9, 95% CI =3.33,14.3,). This is comparable to the study conducted in Addis Ababa which is two times (AOR=1.8, 95%CI=1.79, 3.69) (9). These findings suggest that when health professionals devote

more time to patients, the delivery of key SIC components significantly increases as evidenced by a study conducted in south eastern Ethiopia (5)**.**

Participants counseled one day before surgery were three times more likely to receive the six or more components of SIC compared to those counseled immediately before surgery (AOR=3.27, 95%CI=1.5, 7.11, P=0.003). This finding is in line with a study conducted in Hawassa as 80% of patients who received SIC immediately before surgery were less likely to receive the six or more components of SIC (OR: 0.02; 95% CI: 0.01–0.10) (8). This shows patients counseled earlier before surgery has adequate time to discuss their surgery, thus they are not rushed to undergo surgery with limited information.

Participants who underwent surgery at a specialized hospital were 2.8 times more likely to receive six or more components of SIC than those who went to general hospitals (AOR = 2.85, 95% CI = 1.26, 6.46 P= 0.012). This may be due to specialized hospital often have greater staffing and resources, including students who can provide additional information about the surgery.

Participants who were counseled by a senior physician are 3 times more likely to receive six or more components of SIC than those who didn’t know who counseled them (AOR = 3.185, 95% CI = 1.21, 8.38, P = 0.019). This might be senior physicians tend to offer more comprehensive explanations about the procedure and hold primary responsibility for their patients' overall health (6).

## Limitations of the study

Recall bias was a concern as patients were interviewed after surgery. As it was cross-sectional study, doesn’t show the cause and effect relationship. Finally, this study was conducted only in Mekelle city public hospitals, Tigray, the generalizability of its results to other zones of Tigray may be limited.

# Conclusion

This study showed that majority of participants who underwent both elective and emergency surgery did not receive comprehensive information during the SIC process in the study hospitals. The absence of thorough and standardized pre-operative counseling for surgical procedures undermines, the quality of care patients receive and ability of healthcare facilities to meet patient expectations and information needs. Educational level, timing of SIC delivery, qualification of counselor who took SIC, hospital type, and counselor time spent on informed consent had significant effect on receipt of six or more components of SIC.

To improve SIC delivery it is suggested that health professionals; create rapport with patient, spend more time during counseling, and deliver it early.

**Availability of data and materials**

All data are in the manuscript and/or supporting information file.

**Competing interest**

The authors declare that they have no competing interests

**Funding**

No funding’s

**Author’s contribution**

FA – developed the proposal, designed and participated in data collection, conducted the data analysis and interpretation, developed the first draft, revised subsequent drafts, and wrote the manuscript. HK- advised on the conception of the research proposal, data analysis, interpretation, reviewed and commented on successive drafts. DB- advised on the development of the research proposal, data analysis, interpretation, reviewed and commented on successive drafts. BT – participated in the data collection, reviewing and editing the original draft. FM – participated in the data analysis, and data curation. MA participated in the data analysis, and data curation. All authors reviewed and approved the final draft of the manuscript.

**Authors information**

FG (MSc. in adult health Nursing) lecturer in Aksum University; HK (PhD) Associate professor in Mekelle University; DB (MSc. in adult health Nursing) Assistant Professor in Mekelle University; BT (MSc. in adult health Nursing) lecturer in Adigrat University; FM (MSc. in adult health Nursing) lecturer in Gambella teachers’ education and health science college. MA (MSc. in adult health Nursing) lecturer in Adigrat University.

## Abbreviations

AOR – Adjusted Odds Ratio; CRD- Crude; Odds Ratio; E.C- Ethiopian calendar; EMA- Ethiopian Medical Association; G.C- Gregorian calendar ; GH- General Hospital; IRB- Institutional Review Board; MCC- Motivated Competent Compassionate; MGH- Mekelle General Hospital; OR – Odds Ratio; SIC- Surgical Informed Consent; SPSS: Statically Package Software for Social Science; UK – United Kingdom.

## Acknowledgement

First of all, I would like to express my sincere gratitude to the study participants for their willingness to participate in the study, and to my data collectors and supervisors for their facilitation, organization and collection of the data throughout the study period.

Finally, I extend my sincere thanks to Aksum University for providing me with the opportunity to expand my knowledge and refine my skills. I am also grateful to Mekelle University, College of Health Sciences, School of Nursing for their support in completing my thesis report.

# Reference


1. Farmer L, Lundy A. Informed consent: Ethical and legal considerations for advanced practice nurses. The Journal for Nurse Practitioners. 2017;13(2):124-30.
2. Fields LsI. The Royal College of Surgeons of England. Consent: supported decision making. A guide to good practice. London: The Royal College of Surgeons;. 2016.
3. Leclercq WK, Keulers BJ, Scheltinga MR, Spauwen PH, van der Wilt G-J. A review of surgical informed consent: past, present, and future. A quest to help patients make better decisions. World journal of surgery. 2010;34(7):1406-15.
4. Ochieng J, Buwembo W, Munabi I, Ibingira C, Kiryowa H, Nzarubara G, et al. Informed consent in clinical practice: patients' experiences and perspectives following surgery. BMC research notes. 2015;8(1):1-6.
5. Negash W, Assefa N, Baraki N, Wilfong T. Practice and factors associated with informed consenting process for major surgical procedures among health-care workers, south eastern Ethiopia. International Journal of General Medicine. 2021:7807-17.
6. Rajesh D, Singh A, Chopra M, Singh G, Venkteshan M, Bhardwaj A, et al. Patients awareness, attitude, understanding and perceptions towards legal nature of informed consent. J Indian Acad Forensic Med. 2013;35(1):0971-3.
7. Ogundiran TO, Adebamowo CA. Surgeons' opinions and practice of informed consent in Nigeria. Journal of medical ethics. 2010;36(12):741-5.
8. Teshome M, Wolde Z, Gedefaw A, Tariku M, Asefa A. Surgical informed consent in obstetric and gynecologic surgeries: experience from a comprehensive teaching hospital in southern Ethiopia. BMC medical ethics. 2018;19(1):1-9.
9. Kebede E, Tasew T. Surgical Informed Consent in Clinical Practice: Patients' Perspective Undergoing Cesarean Section at Three Teaching Hospitals in Addis Ababa, Ethiopia. Ethiopian Journal of Health Sciences. 2023;33(4).
10. Gebregziabher M, Amdeselassie F, Esayas R, Abebe Z, Silvia H, Teklehaimanot AA, et al. Geographical distribution of the health crisis of war in the Tigray region of Ethiopia. BMJ global health. 2022;7(4):e008475.
11. Sheikhtaheri A, Farzandipour M. Factors associated with quality of informed consent in patients admitted for surgery: an Iranian study. AJOB Primary Research. 2010;1(4):9-16.
12. Convie L, Carson E, McCusker D, McCain R, McKinley N, Campbell W, et al. The patient and clinician experience of informed consent for surgery: a systematic review of the qualitative evidence. BMC Medical Ethics. 2020;21(1):1-17.
13. MOH E. Federal Democratic Republic of Ethiopia Ministry of Health Health Sector Development Program IV October 2010 Contents. October 2010. 2014.
14. Cawich SO, Barnett AT, Crandon IW, Drew SD, Gordon-Strachan G. From the patient's perspective: is there a need to improve the quality of informed consent for surgery in training hospitals? The Permanente Journal. 2013;17(4):22.

Manuscript
Click here to access/download;Manuscript;munscript - 1.docx

**Research article**

# Surgical informed consent practice and associated factors among adult postoperative patients in public hospitals of mekelle, tigray, ethiopia 2023/2024. Cross sectional

**Authors**

Fiseha Abadi Gebreanenia[1*, #a], Hailemarim Berhe Kahsay[2, #b], Desta Siyoum Belay[2, #b], Binyam Gebrehiwet Tesfay[1, #a], Fissha Brhane Mesele[3,#b] , Mamush Gidey Abrha[4,#c]

[1] Aksum University, college of health science, department of nursing, Axum, Ethiopia

[2] Mekelle University, College of health science, School of nursing, Mekelle, Ethiopia

[3] Gambella teachers' education and health Science College

[4] Adigrat University, college of medicine and health science, Department of nursing, Adigrat, Ethiopia

[#a] current address: Aksum, Northern Ethiopia

[#b] current address: Mekelle, Northern Ethiopia

[#c] current address: Adigrat, Northern Ethiopia

*Fiseha@aku.edu.et (FA)

## Abstract

**Background**

Substantial weaknesses and omissions of surgical informed consent are evident and the current elements of the surgical informed consent process are largely neglected in daily practice. This study aimed to assess surgical informed consent practice and associated factors among adult postoperative patients in public hospitals of Mekelle, Ethiopia.

**Methods**

Institution based cross-sectional study was conducted among 314 adult postoperative patients in public hospitals of Mekelle, Ethiopia. Descriptive, bivariate and multivariable logistic regression analyses were performed using statistical package for social science version 27.

**Result**

Only 35.8% (CI 95%, 30.6, 41) of the respondents were identified to have received the recommended (6 or more) components of surgical informed consent. Educational level [AOR 5.76 (1.02, 32.6)], timing of surgical informed consent delivery [AOR 3.27 (1.5, 7.11)], qualification of counselor who took surgical informed consent them [AOR3.185 (1.21, 8.38)], hospital type [AOR 2.85 (1.26, 6.46)], and duration of counseling [AOR 6.9 (3.33, 14.3)] were statistically significant factors.

**Conclusion**

Majority of participants did not receive comprehensive information during the surgical informed consent process in the study hospitals. To improve the delivery it is suggested that health professionals; create rapport with patient, spend more time during counseling, and deliver it early.

# Introduction

Informed consent is a process that includes providing the patient with information about his or her health status, diagnosis, treatment plans, treatment options, and prognosis to make voluntary and informed decision about his/her treatment(1). As part of Informed consent, surgical informed consent (SIC) is the process whereby patients are informed of all the necessary information about the general health, type of surgery, subsequent treatment plans, risk and alternatives. Informed consent is built by competent patient to make decision about one's surgical procedure with enough information which is clear, understandable and valid((2, 3).

Surgical informed consent is improperly practiced and violated in many occasions; this is mostly due to lack of health care provider awareness and experience with SIC, heavy workload and negligence of health care providers, and lack of a standard consent form (4, 5). While evidence suggests that every surgical patient should be informed by the performing healthcare provider about the proposed surgery before signing a consent form, this isn't always the case in clinical settings, particularly in developing countries (6).

A study in Italy revealed that 84.5% of the participants either personally or through a delegate signed the informed consent. Most attention was given to the diagnosis and the type of surgical procedure, which was communicated respectively to 92.8 and 88.2% of the patients (10).

Another study conducted in Nigeria among surgeons revealed that, 54.9% of the surgeons agreed that sufficient information is not provided to patients while obtaining their consent for surgical procedures (7).

In Ethiopia a cross-sectional study conducted on Surgical informed consent using 13 components of SIC showed that, significant proportion (73.5%) of the respondents hasn't received at least six

of the 13 components of SIC suggested by the investigators which is unacceptable (12). Similar study conducted in Addis Ababa among patients undergone caesarian section 44.9% received the minimum required components of surgical informed consent (8, 9). This could be severe in war torn Tigray as Only 9.7% of health centers, 43.8% of general hospitals and 21.7% of primary hospitals are functional and many health professionals fled out of the region (10).

If adequate information is not provided, the legal consequences could potentially involve lawsuit, professional licensure actions, and negative accreditation implications for the provider and the institution wherein the treatment occurred (1).

According to previous studies factors like educational level, type of surgery, time of counseling, schedule of surgery and duration of counseling were significantly associated with SIC practice (4, 8, 9, 11).

To alleviate the problem the ministry of health of Ethiopia has launched and formulated strategies for healthcare ethics education for students and healthcare professional’s development, this program is called ‘Motivated, Competent and Compassionate Health Workforce’ (12). However, few health care professionals were trained and its applicability on the ground has been poor (13).

While several studies in Ethiopia have examined patients' knowledge and perception of surgical informed consent, few have investigated the actual practice of informed consent as experienced by patients. Additionally, existing research has primarily focused on specific specialties and among healthcare professionals. In addition to this there is no study conducted in the study area and in the region as well. This study aims to address this gap by investigating surgical informed consent practice and its associated factors from patient perspective among adult surgical patients in

Mekelle public hospitals. This study aimed to address the gap in research by determining the prevalence and associated factors of workplace violence against nurses in Mekelle, Ethiopia.

This study's findings will benefit participants by raising their awareness of legal proceedings regarding their treatment. Healthcare providers will gain insight into their current SIC practices and can use this information to improve their services, ultimately minimizing litigation and medico-legal issues. Regulators and ethical committees can utilize this study's findings to assess current SIC practices and identify potential gaps. Additionally, the research can serve as valuable baseline data for future studies with similar interests.

# Methods and materials

## Study area

The study was conducted in public hospitals found in Mekelle, the capital city of Tigrai regional state of Ethiopia. The city lies 780km north of Ethiopian capital Addis Abeba, at 2254m elevation above sea level. Administratively Mekelle is considered as special zone, which is divided into seven sub cities. Mekelle is the economic, cultural, and political hub of Tigray. The city has 1 referral hospital, 2 general hospitals 1 primary hospital and 9 health centers. Operative services are available at the city's referral and general hospitals. The number of major surgeries done monthly on average in Mekelle is around 1001.

## Study period

The study was conducted from December 20th 2023 to January 20th 2024 G.C.

## Study design

Institution based cross-sectional study design was employed to conduct the study.

## Population

### Source population

All adult postoperative patients in public hospitals of Mekelle during data collection.

### Study population

All adult postoperative patients admitted to public hospitals of Mekelle during the data collection period.

## Eligibility criteria

### Inclusion criteria

- All admitted adult postoperative patients were included in the study.

### Exclusion criteria

- The study excluded patients who were not fully conscious during data collection or whose relatives who signed the consent were unavailable.
- Patients who had undergone repeated surgery were excluded to avoid confounding between the two informed consent processes.

## Sample size determination

The actual sample size for the study was determined using single population proportion formula taking 26.5%(patients who received at least six components of SIC) proportion taken from a research conducted in Hawassa (8); with 95% confidence interval (1.96); $\alpha$=0.05 ;5% marginal of error (5%).

$$n = \frac{(Z\alpha/2)2 * p\ (1-p)}{d^2}$$

Where:-

n= required sample size

z= critical value at 95% CI (1.96)

p= prevalence rate, 26.5% i.e. 0.265

Margin of error (d) to be 5% (d = 0.05)

$$n = \frac{(1.96)^2 * 0.265(1-0.265)}{(0.05)^2} \quad n = 299$$

By adding 5% (15) of non-response rate the final sample size was **314.**

## Sampling procedure and technique

All public hospitals in Mekelle city offering surgical services were selected for the study. Within each hospital, the sample size for each surgical specialty was proportionally allocated based on the average monthly number of major surgeries performed (N). Participants were chosen using systematic random sampling with a predetermined sampling interval (K) which is three. The K value was determined by dividing the number of average surgeries done in all public hospital to the sample size. In selected hospitals and wards, participants were interviewed based on their sequence of operations done for each ward every K intervals. *(figure1)*

## Data collection method and tools

Data was collected by four nursing students and one graduated BSc nurse supervisor with no affiliation to the study hospitals, and it was collected using an interviewer administered questionnaire. The questionnaire was developed from various sources (2, 8, 9, 14). It included three main sections: socio-demographic characteristics, service-related characteristics, and thirteen essential components of SIC based on recommendation from ‘Royal College of Surgeons standards of informed consent practices for surgical procedures’. Data collection spanned one month, with interviews and document reviews conducted daily on working days, starting from the first postoperative day and continued until patient discharge.

# Variables

## Dependent variables

- Surgical informed consent practice

## Independent variable

- Socio demographic characteristic (age, sex, marital status, educational level etc...
- Service related characteristics
  - schedule of surgery (emergency/elective),
  - Type of hospital(general, specialized)
  - Timing of SIC delivery (immediately before surgery, one day before surgery...)
  - Type of surgery(surgical, gyn/obs, orthopedic)
  - Duration of counseling( <5minutes, >5minutes)
  - Qualification of health professionals (senior physician, resident/GP, Nurse...)

# Operational definitions

**Practice of SIC:** is defined as receipt of six or more (the minimum recommendation) components from the total thirteen components of the SIC (8, 9).

- **Thirteen components of SIC**

1. Respondent/respondent's family was requested for surgical informed consent.
2. Respondent/respondent's family signed on a surgical informed consent form.
3. Respondent was informed why the surgery will be performed (indication of surgery).

4. Respondent was informed the expected time the surgery will take.
5. Respondent was informed about presence/absence of alternative treatment options.
6. Respondent was informed about type of anesthesia to be used.
7. Respondent was given counseling aids which assist in decision making.
8. Respondent was informed about potential complication/s which may arise.
9. Respondent was informed about consequences of refusing the proposed surgery.
10. There was a favorable environment to say “No” to the proposed surgery.
11. Respondent was given adequate time for decision to sign on the informed consent form.
12. Respondent given opportunity to choose from anesthesia options.
13. Respondent was given an opportunity to ask question.

## Data analysis and management

The data was coded and entered in to Epi Info version 7.2. After cross-checking for consistency and accuracy, the data was cleaned and exported to SPSS version 27 for statistical analysis. Descriptive statistics were performed, Frequency distributions and percentages were employed for categorical variables and the results were summarized using tables, text, and figures.

Both bivariable and multivariable logistic regression analyses were used to determine the association between independent variable and dependent variable. In Bivariable analysis variables having p value less than 0.2 were entered in to multivariable logistic regression to assess statistical association between the outcome variable and independent variables. In multivariable logistic regression, p value $<0.05$ with 95% confidence interval (CI) were considered statistically significant. The Hosmer- Lemeshow goodness-of-fit model coefficients tests procedure was used

to test for model fitting. The assumptions of logistic regression was checked before proceeding to regression.

## Data quality assurance

To ensure data quality, a standard questionnaire relevant to this study was obtained from published journal articles. The English questionnaire was translated into Tigrigna, and the Tigrigna version was then back-translated into English to maintain consistency. However, to meet the objectives of the study, Pretest were done in 5% of sample size in Axum Referral Hospital and Kidiste Mariyam Hospital one week before scheduled data collection day to improve the tool. To standardize data collection techniques, the data collectors received a one-day training session covering the questionnaire's principles, ethical considerations, and question meanings. The supervisor and principal investigator closely supervised the data collectors during data collection. Each completed questionnaire was immediately checked for errors, completeness and legibility.

## Ethical considerations

An ethical clearance letter was obtained from the Institutional Review Board (IRB) of the College of Health Sciences at Mekelle University. Permission to conduct the study was also granted by the Tigray Regional Health Bureau. Participation was voluntary and anonymous. After obtaining full written informed consent from each respondent, confidentiality was assured throughout the data collection period and beyond the study's completion. Individuals who were unwilling to participate, either initially or at any point during the interview, were free to withdraw without consequence. The study design did not involve any risks of danger or hazardous procedures that could harm participants. All methods were performed in accordance with declarations of Helsinki.

## Dissemination of the result

A research report will be submitted and presented to Mekelle University school of Nursing. The results of the study will be communicated with Mekelle health office and each hospital and Tigray regional health bureau. Efforts will be made to publish the results in a peer reviewed scientific journal and make presentations at seminars, workshops and scientific conferences.

# Result

In this study, a total of 310 out of 314 samples were included, yielding a response rate of 98.7%.

## Socio demographic characteristics of study participants

Out of the 310 post-operative patients interviewed, 160 (51.6%) were males. From the total respondents 140 (45.2%) were between the ages of 26 and 40 years. Out of the total study participants (87.4%) were Orthodox followers and (72.3%) of them were married. *(Table 1)*

**Table 1: Socio-demographic characteristics of respondents to assess SIC practice among adult postoperative patients in public hospitals of Mekelle, Ethiopia 2023/2024 (n=310)**

| Variable | Category | Frequency | % |
|---|---|---|---|
| Sex | Male | 160 | 51.6 |
| | Female | 150 | 48.4 |
| | Total | 310 | 100 |
| Religion | Orthodox | 271 | 87.4 |
| | Muslim | 36 | 11.6 |
| | Catholic | 2 | 0.6 |
| | Protestant | 1 | 0.3 |
| | Total | 310 | 100 |
| Marital status | Married | 224 | 72.3 |
| | Single | 49 | 15.8 |
| | Divorced | 19 | 6.1 |
| | Widowed | 15 | 4.8 |
| | Total | 310 | 100 |
| Educational level | Unable to read and write | 92 | 29.7 |
| | Able to read and write | 47 | 15.2 |
| | Primary(1-8) | 54 | 17.4 |
| | Secondary (9-12) | 64 | 20.6 |

| | | | |
|---|---|---|---|
| | Diploma | 27 | 8.7 |
| | Degree | 23 | 7.4 |
| | Master and above | 3 | 1 |
| | Total | 310 | 100 |
| Occupation | Government employee | 45 | 14.5 |
| | Private employee | 18 | 5.8 |
| | Farmer | 114 | 36.8 |
| | Housewife | 72 | 23.2 |
| | Private business | 23 | 7.4 |
| | Daily laborer | 13 | 4.2 |
| | Other | 25 | 8.1 |
| | Total | 310 | 100 |
| Residence | Urban | 146 | 47.1 |
| | Rural | 164 | 52.9 |
| | Total | 310 | 100 |

## Service related characteristics of the respondents

Nearly half of the patients (49.4%) who underwent surgery were unaware of qualifications of the healthcare provider who counseled them about surgical informed consent (SIC). The counseling session duration for most patients (77.7%) was less than 5 minutes. Over half of the surgeries were emergency procedures (59.4%). Out of the total surgeries done (43.5%) were general surgery. Additionally, out of the total surgeries in this study 194(62.6%) were performed at Ayder Specialized Hospital. *Table 2*

**Table 2: Service related characteristics of the respondents to assess SIC practice among adult postoperative patients in public hospitals of Mekelle, Ethiopia 2023/2024 (n=310)**

| Variable | Category | Frequency | Percent |
|---|---|---|---|
| Duration of counseling | <05 minutes | 241 | 77.7 |
| | >05 minutes | 69 | 22.3 |
| | Total | 310 | 100 |
| Qualification of counselor who took surgical informed consent | Senior physician | 68 | 2 |
| | Resident physician | 29 | 9.4 |
| | Intern or GP | 5 | 1.6 |
| | Nurse | 32 | 10.3 |
| | Midwife | 23 | 7.4 |
| | Did not know | 153 | 49.4 |
| | Total | 310 | 100 |
| Schedule of surgery | Elective | 126 | 40.6 |
| | Emergency | 184 | 59.4 |
| | Total | 310 | 100 |
| Type of hospital | Specialized | 194 | 62.6 |
| | General | 116 | 37.4 |
| | Total | 310 | 100 |
| Type of anesthesia | General | 137 | 44.2 |
| | Spinal | 173 | 55.8 |
| | Total | 310 | 100 |
| Timing of counseling for informed consent | The day before date of surgery | 109 | 35.2 |
| | On the day of surgery | 58 | 18.7 |
| | Immediately before surgery | 126 | 40.6 |
| | On the operation table | 17 | 5.5 |
| | Total | 310 | 100 |
| Type of surgery | General surgery | 135 | 43.5 |
| | Orthopedic | 64 | 20.6 |
| | Gynecology/obstetric | 111 | 35.9 |

| | | |
|---|---|---|
| Total | 310 | 100 |

## Components of surgical informed consent received by respondents

Almost all (99%) of the respondents or their family members signed an informed consent form. Most respondents (87.7%) were informed about the indication for the surgery. However, only 8.4% of respondents were informed about the expected surgery duration, and very few respondents (1%) were given a chance to choose their anesthesia. More than half (55.5%) of respondents received information about potential complications during the procedure. *Table 3*

**Table 3: Essential components of surgical informed consent received by respondents to assess SIC practice among adult postoperative patients in public hospitals of Mekelle, Ethiopia 2023/2024 (n=310)**

| **Variables** | **Responses , n, %** | | |
|---|---|---|---|
| | Yes | No | Do not remember |
| Respondent/respondent’s family was requested for surgical informed consent | 303(97.7) | 5(1.6) | 2(0.6) |
| Respondent/respondent’s family signed on an surgical informed consent form | 307(99) | 3(1) | _ |
| Respondent was informed why the surgery will be performed (indication of surgery) | 272(87.8) | 33(10.6) | 5(1.6) |
| Respondent was informed the expected time the surgery will take | 26(8.4) | 273(88.1) | 11(3.5) |
| Respondent was informed about presence/absence of alternative treatment options | 47(15.2) | 254(81.9) | 9(2.9) |
| Respondent was informed about type of anesthesia to be used | 19(6.1) | 275(88.7) | 16(5.2) |
| Respondent was given counseling aids which assist in decision making | 140(45.2) | 163(52.6) | 7(2.2) |
| Respondent was informed about potential complication/s which may arise | 203(65.5) | 100(32.3) | 7(2.2) |
| Respondent was informed about consequences of refusing the proposed surgery | 179(57.7) | 125(40.3) | 6(2) |
| There was a favorable environment to say “No” to the proposed surgery | 58(18.7) | 248(80) | 4(1.3) |

| | | | |
|---|---|---|---|
| Respondent was given adequate time for decision to sign on the informed consent form | 164(52.9) | 139(44.8) | 7(2.3) |
| Respondent was given opportunity to choose from anesthesia options | 3(1) | 298(96.1) | 9(2.9) |
| Respondent was given an opportunity to ask question | 127(41) | 179(57.7) | 4(1.3) |

## Receipt of six or more components of surgical informed consent

Based on the total numbers of the components of SIC respondents received before their surgery, out of the total thirteen components 35.8% (CI =95%, 30.6, 41)of the respondents were identified to have received six or more (the minimum recommendation) components of surgical informed consent. *Figure 3*

## Factors associated with receipt of six or more components of surgical informed consent

In bi-variable regression analysis variables like educational level, occupation, sex, type of anesthesia, type of surgery, schedule of surgery, timing of SIC delivery, health care provider who took SIC, hospital type, and counselor time spent on informed consent were significant at $P= <0.2$, thus they were selected for multi-variable regression analysis. Following multi-variable regression analysis variables like educational level, timing of SIC delivery, Qualification of counselor who took SIC, hospital type, and counselor time spent on informed consent were significant at $P<0.05$. *Table 4*

**Table 4; Bi-variables and multi-variable analysis to assess factors associated with receipt of six or more components of SIC among adult postoperative patients in public hospitals of Mekelle, Ethiopia 2023/2024 n=310**

| Variable | Category | Received 6 or more components of SIC | | COR (95%CI) | AOR(95% CI) | P-value |
|---|---|---|---|---|---|---|
| | | Yes | No | | | |
| Educational level | able to read and write | 16 | 30 | 1.47(0.69,3.15) | 2.29(0.84,6.2) | 0.105 |
| | Primary(1-8) | 19 | 38 | 1.38(0.67,2.82 | 2.1(0.75,5.9) | 0.157 |
| | Secondary (9-12) | 19 | 41 | 1.28(0.63,2.64) | 2.5(0.68,8.2) | 0.129 |
| | Diploma | 15 | 13 | 3.18(1.33,7.62) | 5.1(0.93,28.15) | 0.06 |
| | Degree and above | 17 | 8 | 5.86(2.25,15.27) | **5.76(1.02,32.6)*** | **0.047*** |
| | Unable to read and write | 25 | 69 | Ref. | Ref. | |
| Duration of counseling | <05 minutes | 61 | 180 | Ref. | Ref. | |
| | >05 minutes | 50 | 19 | 7.765(4.25,14.19) | **65.9(3.33,14.3)*** | **<0.001*** |
| Qualification of counselor who took SIC | Did not know | 48 | 105 | Ref. | Ref. | |
| | Senior physician | 39 | 29 | 2.94(1.63,5.3) | **3.18(1.21,8.38)*** | **0.019*** |
| | Resident physician, Intern or GP | 10 | 24 | 0.91(0.4,2) | 0.445(0.15,1.35) | 0.153 |
| | Nurse | 9 | 23 | 0.856(0.37,1.99) | 1.89(0.59,6.1) | 0.287 |
| | Midwife | 5 | 18 | 0.6(0.21,1.73) | 1.34(0.3,6) | 0.704 |
| Type of hospital | General | 31 | 85 | Ref. | Ref. | |
| | Specialized | 80 | 114 | 1.92(1.16,3.17) | **2.85(1.26,6.46)*** | **0.012*** |
| Timing of counseling for informed consent | Immediately before surgery | 35 | 108 | Ref. | Ref. | |
| | The day before date of surgery | 59 | 50 | 3.64(2.13,6.22) | **3.27(1.5,7.11)*** | **0.003*** |
| | On the day of surgery | 17 | 41 | 1.28(0.65,2.53) | 0.86(0.36,2.1) | 0.751 |
| Occupation | Daily laborer | 4 | 9 | Ref. | Ref. | |
| | Private employee | 6 | 12 | 1.125(0.243,5.2) | 0.51(0.79,3.3) | 0.481 |
| | Farmer | 37 | 77 | 1.08(0.31,3.74) | 1.09(0.21,5.53) | 0.919 |
| | Housewife | 16 | 56 | 0.643(0.175,2.36) | 0.99(0.17,5.6) | 0.988 |
| | Private business | 5 | 18 | 0.625(0.134,2.9) | 0.57(0.85,3.84) | 0.564 |

| | | | | | | |
|---|---|---|---|---|---|---|
| | Government employee | 29 | 16 | 4.08(1.08,15.37) | 1.59(0.24,10.5) | 0.629 |
| | Other | 14 | 11 | 2.86(0.69,11.82) | 2.34(0.43,12.8) | 0.326 |
| Sex | Female | 48 | 102 | Ref. | Ref. | |
| | Male | 63 | 97 | 1.38(0.8,2.2) | 0.92(0.37,2.3) | 0.86 |
| Schedule of surgery | Elective | 51 | 75 | 1.4(0.88,2.25) | 1.01(0.49,2.07) | 0.977 |
| | Emergency | 60 | 124 | Ref. | Ref. | |
| Type of surgery | General surgery | 53 | 82 | Ref. | Ref. | |
| | Orthopedic | 30 | 34 | 1.37(0.75,2.5) | 1.28(0.55,2.98) | 0.565 |
| | Gynecology/obstetric | 28 | 83 | 0.52(0.3,0.9) | 0.76(0.24,2.4) | 0.641 |
| Type of anesthesia | General | 59 | 78 | 1.76(1.1,2.81) | 1.07(0.51,2.24) | 0.854 |
| | Spinal | 52 | 121 | Ref. | Ref. | |

***= Statistically significant with p- value < 0.2** **1=Reference group of the variable**

****=Statistically significant with p- value <0.05**

# Discussion

The receipt of six or more (the minimum recommendation) components of SIC in this study is (35.8%) with (CI 95%, 30.6, 41), which is slightly higher than the study conducted at Hawassa (26.5%) (8). This difference might be attributable to the study area covered, which exposes patients to various health professionals and different working environments. This is because this study included all public hospitals and post-op wards, whereas the latter study was exclusively conducted in the gynecology/obstetrics department of a specific hospital. On the other hand, it is lower than the study conducted in Addis Ababa (44.9%) (9). This disparity is likely due to decreased staff morale in helping patients in the study area, possibly stemming from a lack of incentives, service delivery arrangements, resource capacities and increased workload (5).

A multi-variable regression analysis identified several factors associated with receipt of six or more components of SIC. Participants with a degree or higher educational level were 5.7 times more likely to receive 6 or more components than those who were unable to read or write (AOR=5.76, 95%CI=1.02, 32.6, P=0.047)**.** These findings are in line with a study conducted in Iran, and Addis Ababa (AOR=2.4, 95%CI=1.81, 11.32) (9, 11). This difference might be attributable to the fact that educated participants tend to ask more questions. This, in turn, led them to receive more information about the proposed surgery.

Participants counseled for more than 5 minutes were almost seven times more likely to receive the 6 or more components of SIC than those counseled for less than 5 minutes (AOR= 6.9, 95% CI =3.33,14.3,). This is comparable to the study conducted in Addis Ababa which is two times (AOR=1.8, 95%CI=1.79, 3.69) (9). These findings suggest that when health professionals devote

more time to patients, the delivery of key SIC components significantly increases as evidenced by a study conducted in south eastern Ethiopia (5)**.**

Participants counseled one day before surgery were three times more likely to receive the six or more components of SIC compared to those counseled immediately before surgery (AOR=3.27, 95%CI=1.5, 7.11, P=0.003). This finding is in line with a study conducted in Hawassa as 80% of patients who received SIC immediately before surgery were less likely to receive the six or more components of SIC (OR: 0.02; 95% CI: 0.01–0.10) (8). This shows patients counseled earlier before surgery has adequate time to discuss their surgery, thus they are not rushed to undergo surgery with limited information.

Participants who underwent surgery at a specialized hospital were 2.8 times more likely to receive six or more components of SIC than those who went to general hospitals (AOR = 2.85, 95% CI = 1.26, 6.46 P= 0.012). This may be due to specialized hospital often have greater staffing and resources, including students who can provide additional information about the surgery.

Participants who were counseled by a senior physician are 3 times more likely to receive six or more components of SIC than those who didn’t know who counseled them (AOR = 3.185, 95% CI = 1.21, 8.38, P = 0.019). This might be senior physicians tend to offer more comprehensive explanations about the procedure and hold primary responsibility for their patients' overall health (6).

## Limitations of the study

Recall bias was a concern as patients were interviewed after surgery. As it was cross-sectional study, doesn’t show the cause and effect relationship. Finally, this study was conducted only in

Mekelle city public hospitals, Tigray, the generalizability of its results to other zones of Tigray may be limited.

# Conclusion

This study showed that majority of participants who underwent both elective and emergency surgery did not receive comprehensive information during the SIC process in the study hospitals. The absence of thorough and standardized pre-operative counseling for surgical procedures undermines, the quality of care patients receive and ability of healthcare facilities to meet patient expectations and information needs. Educational level, timing of SIC delivery, qualification of counselor who took SIC, hospital type, and counselor time spent on informed consent had significant effect on receipt of six or more components of SIC.

To improve SIC delivery it is suggested that health professionals; create rapport with patient, spend more time during counseling, and deliver it early.

## Declarations

**Ethical approval and consent to participant**

Official ethical clearance letter was obtained from the institutional Review board of the College of Health Sciences of the Mekelle University, after approval the necessary communication were made with chief clinical directors, medical directors and the hospital administrators of each hospital. Written informed consent were obtained from the respective personnel before starting study. Filled questionnaires kept securely and only accessible to the researcher. All methods were performed in accordance with declarations of Helsinki.

**Consent for publication**

Not applicable

**Availability of data and materials**

The datasets used and/or analyzed during the current study are available from the corresponding author on reasonable request.

**Competing interest**

The authors declare that they have no competing interests

**Funding**

No funding's

**Author's contribution**

FA – developed the proposal, designed and participated in data collection, conducted the data analysis and interpretation, developed the first draft, revised subsequent drafts, and wrote the manuscript. HK- advised on the conception of the research proposal, data analysis, interpretation, reviewed and commented on successive drafts. DB- advised on the development of the research proposal, data analysis, interpretation, reviewed and commented on successive drafts. BT –

participated in the data collection, reviewing and editing the original draft. FM – participated in the data analysis, and data curation. MA participated in the data analysis, and data curation. All authors reviewed and approved the final draft of the manuscript.

**Authors information**

FG (MSc. in adult health Nursing) lecturer in Aksum University; HK (PhD) Associate professor in Mekelle University; DB (MSc. in adult health Nursing) Assistant Professor in Mekelle University; BT (MSc. in adult health Nursing) lecturer in Adigrat University; FM (MSc. in adult health Nursing) lecturer in Gambella teachers' education and health science college. MA (MSc. in adult health Nursing) lecturer in Adigrat University.

## Abbreviations

AOR – Adjusted Odds Ratio; CRD- Crude; Odds Ratio; E.C- Ethiopian calendar; EMA- Ethiopian Medical Association; G.C- Gregorian calendar ; GH- General Hospital; IRB- Institutional Review Board; MCC- Motivated Competent Compassionate; MGH- Mekelle General Hospital; OR – Odds Ratio; SIC- Surgical Informed Consent; SPSS: Statically Package Software for Social Science; UK – United Kingdom.


## Acknowledgement

First of all, I would like to express my sincere gratitude to the study participants for their willingness to participate in the study, and to my data collectors and supervisors for their facilitation, organization and collection of the data throughout the study period.

Finally, I extend my sincere thanks to Aksum University for providing me with the opportunity to expand my knowledge and refine my skills. I am also grateful to Mekelle University, College of Health Sciences, School of Nursing for their support in completing my thesis report.

# Reference


1. Farmer L, Lundy A. Informed consent: Ethical and legal considerations for advanced practice nurses. The Journal for Nurse Practitioners. 2017;13(2):124-30.
2. Fields LsI. The Royal College of Surgeons of England. Consent: supported decision making. A guide to good practice. London: The Royal College of Surgeons;. 2016.
3. Leclercq WK, Keulers BJ, Scheltinga MR, Spauwen PH, van der Wilt G-J. A review of surgical informed consent: past, present, and future. A quest to help patients make better decisions. World journal of surgery. 2010;34(7):1406-15.
4. Ochieng J, Buwembo W, Munabi I, Ibingira C, Kiryowa H, Nzarubara G, et al. Informed consent in clinical practice: patients' experiences and perspectives following surgery. BMC research notes. 2015;8(1):1-6.
5. Negash W, Assefa N, Baraki N, Wilfong T. Practice and factors associated with informed consenting process for major surgical procedures among health-care workers, south eastern Ethiopia. International Journal of General Medicine. 2021:7807-17.
6. Rajesh D, Singh A, Chopra M, Singh G, Venkteshan M, Bhardwaj A, et al. Patients awareness, attitude, understanding and perceptions towards legal nature of informed consent. J Indian Acad Forensic Med. 2013;35(1):0971-3.
7. Ogundiran TO, Adebamowo CA. Surgeons' opinions and practice of informed consent in Nigeria. Journal of medical ethics. 2010;36(12):741-5.
8. Teshome M, Wolde Z, Gedefaw A, Tariku M, Asefa A. Surgical informed consent in obstetric and gynecologic surgeries: experience from a comprehensive teaching hospital in southern Ethiopia. BMC medical ethics. 2018;19(1):1-9.
9. Kebede E, Tasew T. Surgical Informed Consent in Clinical Practice: Patients' Perspective Undergoing Cesarean Section at Three Teaching Hospitals in Addis Ababa, Ethiopia. Ethiopian Journal of Health Sciences. 2023;33(4).
10. Gebregziabher M, Amdeselassie F, Esayas R, Abebe Z, Silvia H, Teklehaimanot AA, et al. Geographical distribution of the health crisis of war in the Tigray region of Ethiopia. BMJ global health. 2022;7(4):e008475.
11. Sheikhtaheri A, Farzandipour M. Factors associated with quality of informed consent in patients admitted for surgery: an Iranian study. AJOB Primary Research. 2010;1(4):9-16.
12. Convie L, Carson E, McCusker D, McCain R, McKinley N, Campbell W, et al. The patient and clinician experience of informed consent for surgery: a systematic review of the qualitative evidence. BMC Medical Ethics. 2020;21(1):1-17.
13. MOH E. Federal Democratic Republic of Ethiopia Ministry of Health Health Sector Development Program IV October 2010 Contents. October 2010. 2014.
14. Cawich SO, Barnett AT, Crandon IW, Drew SD, Gordon-Strachan G. From the patient's perspective: is there a need to improve the quality of informed consent for surgery in training hospitals? The Permanente Journal. 2013;17(4):22.

Figure Click here to access/download;Figure;figures.docx

## Schematic presentation of sampling procedure

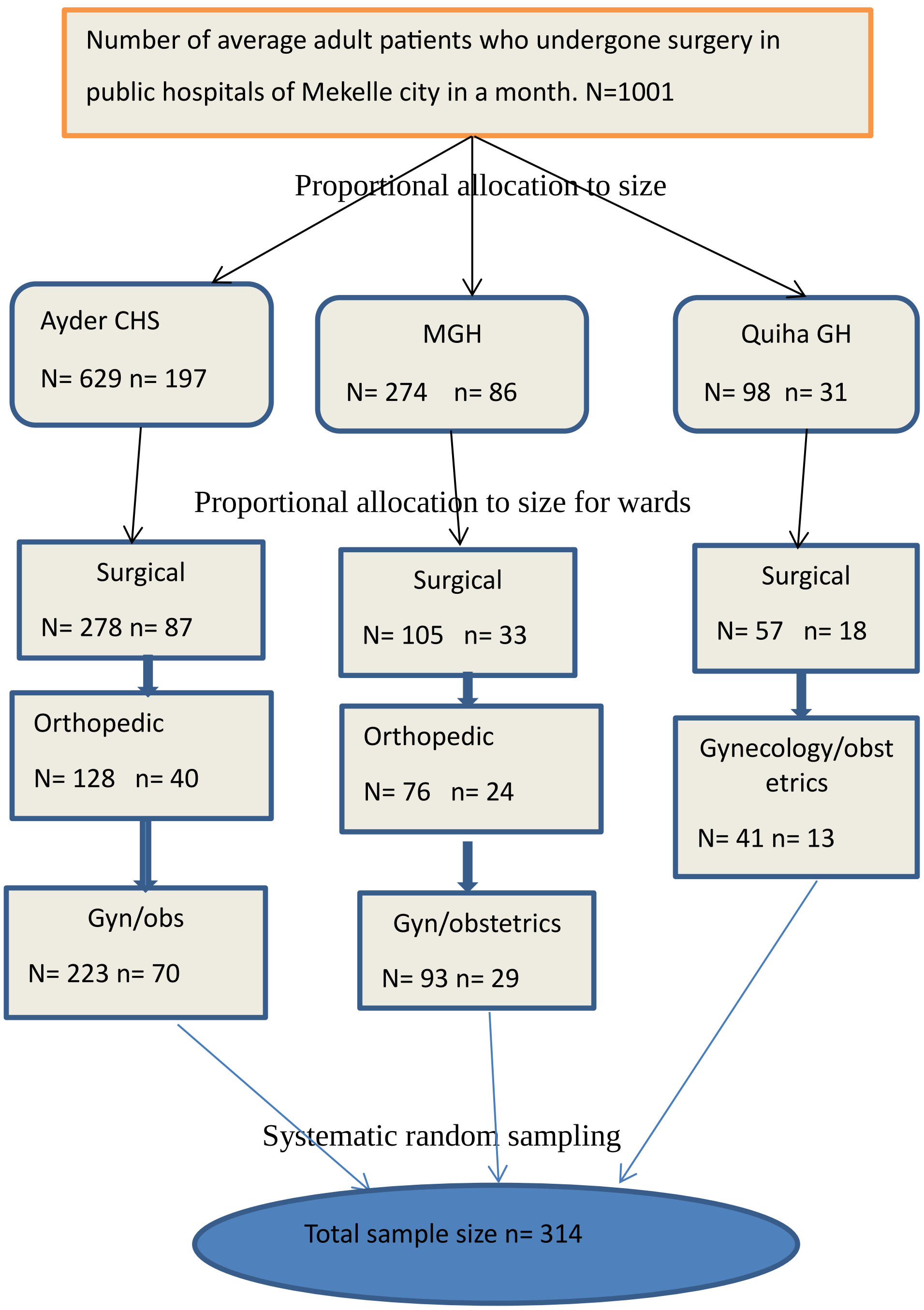


**Figure 1: Schematic presentation of sampling procedure of surgical informed consent practice and associated factors among adult postoperative patients in public hospitals, Meklle, Tigray, Ethiopia 2023/2024.**

*NB*: N ➧ total number of surgeries in a month, n➧ proportional size

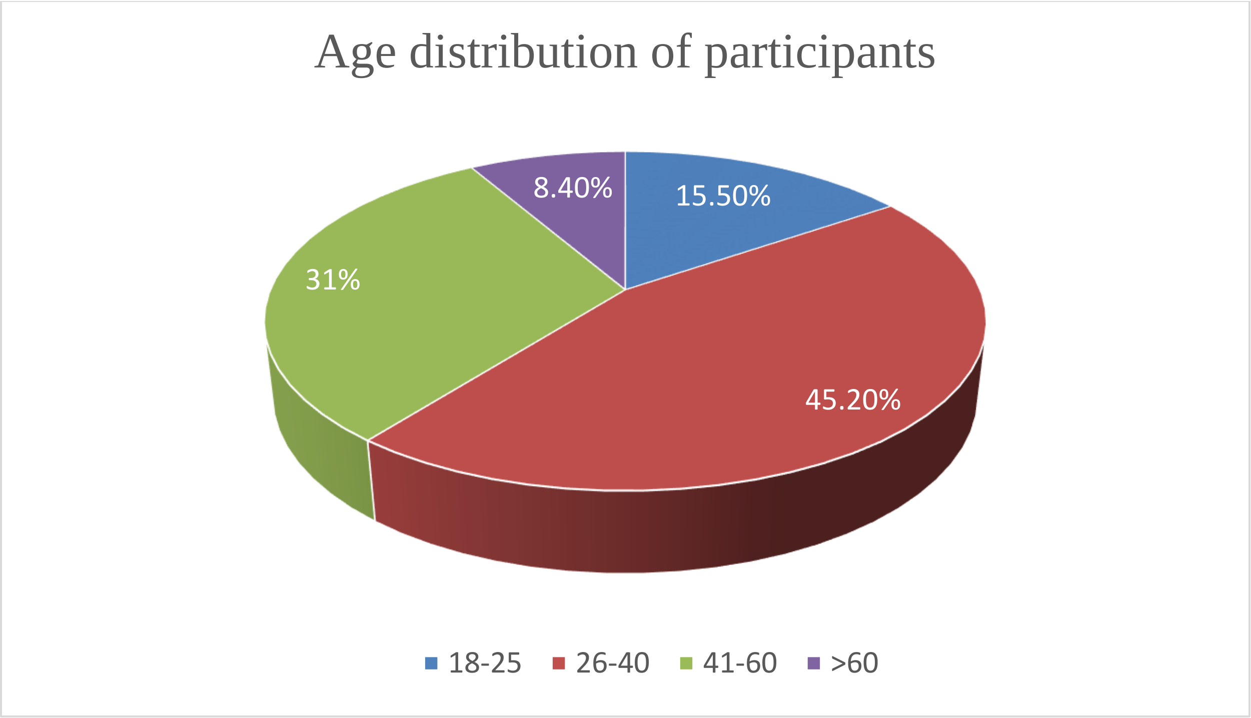


**Figure 2; Age distribution of study participants to assess SIC practice among adult postoperative patients in public hospitals of Mekelle, Ethiopia 2023/2024.**

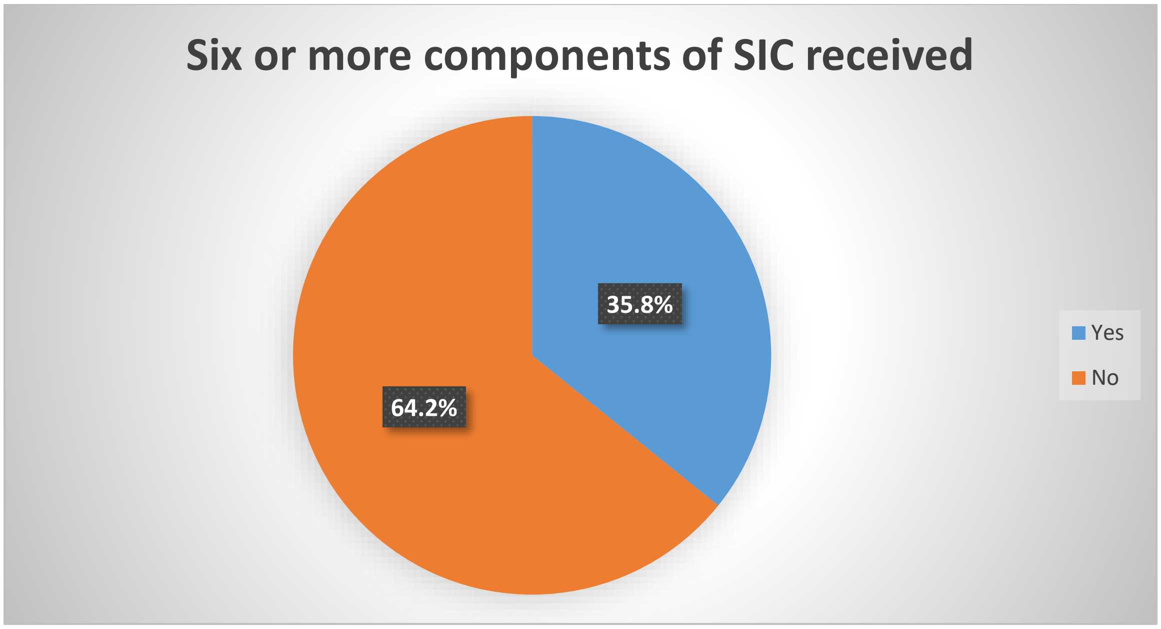


**Figure 3; Receipt of 6 or more components of surgical informed consent, among adult postoperative patients in public hospitals of Mekelle, Ethiopia 2023/2024.**

Click here to access/download
**Supporting Information**
data set.xlsx

**Research article**

# Surgical informed consent practice and associated factors among adult postoperative patients in public hospitals of Mekelle, Tigray, Ethiopia 2023/2024. Cross sectional

**Authors**

Fiseha Abadi Gebreanenia[1*, #a], Hailemarim Berhe Kahsay[2, #b], Desta Siyoum Belay[2, #b], Binyam Gebrehiwet Tesfay[4, #c], Fissha Brhane Mesele[3,#b] , Mamush Gidey Abrha[4,#c]

[1] Aksum University, college of health science, department of nursing, Axum, Ethiopia

[2] Mekelle University, College of health science, School of nursing, Mekelle, Ethiopia

[3] Gambella teachers' education and health Science College

[4] Adigrat University, college of medicine and health science, Department of nursing, Adigrat, Ethiopia

[#a] current address: Aksum, Northern Ethiopia

[#b] current address: Mekelle, Northern Ethiopia

[#c] current address: Adigrat, Northern Ethiopia

*Fiseha@aku.edu.et (FA)

## Abstract

### Background

Substantial weaknesses and omissions of surgical informed consent are evident and the current elements of the surgical informed consent process are largely neglected in daily practice. This study aimed to assess surgical informed consent practice and associated factors among adult postoperative patients in public hospitals of Mekelle, Tigray, Ethiopia.

### Methods

Institution based cross-sectional study was conducted among 314 adult postoperative patients in public hospitals of Mekelle, Tigray, Ethiopia. Descriptive, bivariate and multivariable logistic regression analyses were performed using statistical package for social science version 27.

### Result

Only 35.8% (CI 95%, 30.6, 41) of the respondents were identified to have received the recommended (6 or more) components of surgical informed consent. Educational level [AOR 5.76 (1.02, 32.6)], timing of surgical informed consent delivery [AOR 3.27 (1.5, 7.11)], qualification of counselor who took surgical informed consent them [AOR3.185 (1.21, 8.38)], hospital type [AOR 2.85 (1.26, 6.46)], and duration of counseling [AOR 6.9 (3.33, 14.3)] were statistically significant factors.

### Conclusion

Majority of participants did not receive comprehensive information during the surgical informed consent process in the study hospitals. To improve the delivery it is suggested that health professionals; create rapport with patient, spend more time during counseling, and deliver it early.

# Introduction

Informed consent is a process that includes providing the patient with information about his or her health status, diagnosis, treatment plans, treatment options, and prognosis to make voluntary and informed decision about his/her treatment(1). As part of Informed consent, surgical informed consent (SIC) is the process whereby patients are informed of all the necessary information about the general health, type of surgery, subsequent treatment plans, risk and alternatives. Informed consent is built by competent patient to make decision about one's surgical procedure with enough information which is clear, understandable and valid((2, 3).

Surgical informed consent is improperly practiced and violated in many occasions; this is mostly due to lack of health care provider awareness and experience with SIC, heavy workload and negligence of health care providers, and lack of a standard consent form (4, 5). While evidence suggests that every surgical patient should be informed by the performing healthcare provider about the proposed surgery before signing a consent form, this isn't always the case in clinical settings, particularly in developing countries (6).

A study in Italy revealed that 84.5% of the participants either personally or through a delegate signed the informed consent. Most attention was given to the diagnosis and the type of surgical procedure, which was communicated respectively to 92.8 and 88.2% of the patients (10).

Another study conducted in Nigeria among surgeons revealed that, 54.9% of the surgeons agreed that sufficient information is not provided to patients while obtaining their consent for surgical procedures (7).

In Ethiopia a cross-sectional study conducted on Surgical informed consent using 13 components of SIC showed that, significant proportion (73.5%) of the respondents hasn't received at least six

of the 13 components of SIC suggested by the investigators which is unacceptable (12). Similar study conducted in Addis Ababa among patients undergone caesarian section 44.9% received the minimum required components of surgical informed consent (8, 9). This could be severe in war torn Tigray as Only 9.7% of health centers, 43.8% of general hospitals and 21.7% of primary hospitals are functional and many health professionals fled out of the region (10).

If adequate information is not provided, the legal consequences could potentially involve lawsuit, professional licensure actions, and negative accreditation implications for the provider and the institution wherein the treatment occurred (1).

According to previous studies factors like educational level, type of surgery, time of counseling, schedule of surgery and duration of counseling were significantly associated with SIC practice (4, 8, 9, 11).

To alleviate the problem the ministry of health of Ethiopia has launched and formulated strategies for healthcare ethics education for students and healthcare professional's development, this program is called 'Motivated, Competent and Compassionate Health Workforce' (12). However, few health care professionals were trained and its applicability on the ground has been poor (13).

While several studies in Ethiopia have examined patients' knowledge and perception of surgical informed consent, few have investigated the actual practice of informed consent as experienced by patients. Additionally, existing research has primarily focused on specific specialties and among healthcare professionals. In addition to this there is no study conducted in the study area and in the region as well. This study aims to address this gap by investigating surgical informed consent practice and its associated factors from patient perspective among adult surgical patients in

Mekelle public hospitals. This study aimed to address the gap in research by determining the prevalence and associated factors of workplace violence against nurses in Mekelle, Ethiopia.

This study's findings will benefit participants by raising their awareness of legal proceedings regarding their treatment. Healthcare providers will gain insight into their current SIC practices and can use this information to improve their services, ultimately minimizing litigation and medico-legal issues. Regulators and ethical committees can utilize this study's findings to assess current SIC practices and identify potential gaps. Additionally, the research can serve as valuable baseline data for future studies with similar interests.

# Methods and materials

## Study design, population, area and period

An institution-based cross-sectional study design was employed for this study. The study was conducted in public hospitals located in Mekelle, the capital city of Tigray Regional State, Ethiopia. The average number of major surgeries performed monthly in Mekelle is approximately 1001. Data was collected from December 20, 2023, to January 20, 2024, G.C. The study population included all adult postoperative patients admitted to public hospitals in Mekelle during the data collection period.

## Eligibility criteria

### Inclusion criteria

- All admitted adult postoperative patients were included in the study.

### Exclusion criteria

- The study excluded patients who were not fully conscious during data collection or whose relatives who signed the consent were unavailable.
- Patients who had undergone repeated surgery were excluded to avoid confounding between the two informed consent processes.

## Sample size determination

The actual sample size for the study was determined using single population proportion formula taking 26.5%(patients who received at least six components of SIC) proportion taken from a research conducted in Hawassa (8); with 95% confidence interval (1.96); α=0.05 ;5% marginal of error (5%).

$$n = \frac{(Z\alpha/2)2 * p\,(1-p)}{d^2}$$

Where:-

n= required sample size

z= critical value at 95% CI (1.96)

p= prevalence rate, 26.5% i.e. 0.265

Margin of error (d) to be 5% (d = 0.05)

$$n = \frac{(1.96)^2 * 0.265(1-0.265)}{(0.05)^2} \quad n = 299$$

By adding 5% (15) of non-response rate the final sample size was **314.**

## Sampling procedure and technique

All public hospitals in Mekelle city offering surgical services were selected for the study. Within each hospital, the sample size for each surgical specialty was proportionally allocated based on the average monthly number of major surgeries performed (N). Participants were chosen using systematic random sampling with a predetermined sampling interval (K) which is three. The K value was determined by dividing the number of average surgeries done in all public hospital to the sample size. In selected hospitals and wards, participants were interviewed based on their sequence of operations done for each ward every K intervals. *(figure1)*

## Data collection method and tools

Data was collected by four nursing students and one graduated BSc nurse supervisor with no affiliation to the study hospitals, and it was collected using an interviewer administered

questionnaire. The questionnaire was developed from various sources (2, 8, 9, 14). It included three main sections: socio-demographic characteristics, service-related characteristics, and thirteen essential components of SIC based on recommendation from 'Royal College of Surgeons standards of informed consent practices for surgical procedures'. Data collection spanned one month, with interviews and document reviews conducted daily on working days, starting from the first postoperative day and continued until patient discharge.

# Variables

# Dependent variables

- Surgical informed consent practice

## Independent variable

- Socio demographic characteristic (age, sex, marital status, educational level etc...
- Service related characteristics
    - schedule of surgery (emergency/elective),
    - Type of hospital(general, specialized)
    - Timing of SIC delivery (immediately before surgery, one day before surgery...)
    - Type of surgery(surgical, gyn/obs, orthopedic)
    - Duration of counseling( <5minutes, >5minutes)
    - Qualification of health professionals (senior physician, resident/GP, Nurse...)

# Operational definitions

**Practice of SIC:** is defined as receipt of six or more (the minimum recommendation) components from the total thirteen components of the SIC (8, 9).

- **Thirteen components of SIC**

1. Respondent/respondent’s family was requested for surgical informed consent.
2. Respondent/respondent’s family signed on a surgical informed consent form.
3. Respondent was informed why the surgery will be performed (indication of surgery).
4. Respondent was informed the expected time the surgery will take.
5. Respondent was informed about presence/absence of alternative treatment options.
6. Respondent was informed about type of anesthesia to be used.
7. Respondent was given counseling aids which assist in decision making.
8. Respondent was informed about potential complication/s which may arise.
9. Respondent was informed about consequences of refusing the proposed surgery.
10. There was a favorable environment to say “No” to the proposed surgery.
11. Respondent was given adequate time for decision to sign on the informed consent form.
12. Respondent given opportunity to choose from anesthesia options.
13. Respondent was given an opportunity to ask question.

## Data analysis and management

The data was coded and entered in to Epi Info version 7.2. After cross-checking for consistency and accuracy, the data was cleaned and exported to SPSS version 27 for statistical analysis. Descriptive statistics were performed, Frequency distributions and percentages were employed for categorical variables and the results were summarized using tables, text, and figures.

Both bivariable and multivariable logistic regression analyses were used to determine the association between independent variable and dependent variable. In Bivariable analysis variables having p value less than 0.2 were entered in to multivariable logistic regression to assess statistical

association between the outcome variable and independent variables. In multivariable logistic regression, p value $<0.05$ with 95% confidence interval (CI) were considered statistically significant. The Hosmer- Lemeshow goodness-of-fit model coefficients tests procedure was used to test for model fitting. The assumptions of logistic regression was checked before proceeding to regression.

## Data quality assurance

To ensure data quality, a standard questionnaire relevant to this study was obtained from published journal articles. The English questionnaire was translated into Tigrigna, and the Tigrigna version was then back-translated into English to maintain consistency. However, to meet the objectives of the study, Pretest were done in 5% of sample size in Axum Referral Hospital and Kidiste Mariyam Hospital one week before scheduled data collection day to improve the tool. To standardize data collection techniques, the data collectors received a one-day training session covering the questionnaire's principles, ethical considerations, and question meanings. The supervisor and principal investigator closely supervised the data collectors during data collection. Each completed questionnaire was immediately checked for errors, completeness and legibility.

## Ethical considerations

Official ethical clearance letter was obtained from the institutional Review board of the College of Health Sciences of the Mekelle University, after approval the necessary communication were made with chief clinical directors, medical directors and the hospital administrators of each hospital. Written informed consent were obtained from the respective personnel before starting study. Filled questionnaires kept securely and only accessible to the researcher. All methods were performed in accordance with declarations of Helsinki.

# Result

In this study, a total of 310 out of 314 samples were included, yielding a response rate of 98.7%.

## Socio demographic characteristics of study participants

Out of the 310 post-operative patients interviewed, 160 (51.6%) were males. From the total respondents 140 (45.2%) were between the ages of 26 and 40 years. Out of the total study participants (87.4%) were Orthodox followers and (72.3%) of them were married. *(Table 1)*

**Table 1: Socio-demographic characteristics of respondents to assess SIC practice among adult postoperative patients in public hospitals of Mekelle, Ethiopia 2023/2024 (n=310)**

| Variable | Category | Frequency | % |
|---|---|---|---|
| Sex | Male | 160 | 51.6 |
| | Female | 150 | 48.4 |
| | Total | 310 | 100 |
| Religion | Orthodox | 271 | 87.4 |
| | Muslim | 36 | 11.6 |
| | Catholic | 2 | 0.6 |
| | Protestant | 1 | 0.3 |
| | Total | 310 | 100 |
| Marital status | Married | 224 | 72.3 |
| | Single | 49 | 15.8 |
| | Divorced | 19 | 6.1 |
| | Widowed | 15 | 4.8 |
| | Total | 310 | 100 |
| Educational level | Unable to read and write | 92 | 29.7 |
| | Able to read and write | 47 | 15.2 |
| | Primary(1-8) | 54 | 17.4 |
| | Secondary (9-12) | 64 | 20.6 |
| | Diploma | 27 | 8.7 |
| | Degree | 23 | 7.4 |
| | Master and above | 3 | 1 |
| | Total | 310 | 100 |

| | | | |
|---|---|---|---|
| Occupation | Government employee | 45 | 14.5 |
| | Private employee | 18 | 5.8 |
| | Farmer | 114 | 36.8 |
| | Housewife | 72 | 23.2 |
| | Private business | 23 | 7.4 |
| | Daily laborer | 13 | 4.2 |
| | Other | 25 | 8.1 |
| | Total | 310 | 100 |
| Residence | Urban | 146 | 47.1 |
| | Rural | 164 | 52.9 |
| | Total | 310 | 100 |

## Service related characteristics of the respondents

Nearly half of the patients (49.4%) who underwent surgery were unaware of qualifications of the healthcare provider who counseled them about surgical informed consent (SIC). The counseling session duration for most patients (77.7%) was less than 5 minutes. Over half of the surgeries were emergency procedures (59.4%). Out of the total surgeries done (43.5%) were general surgery. Additionally, out of the total surgeries in this study 194(62.6%) were performed at Ayder Specialized Hospital. *Table 2*

**Table 2: Service related characteristics of the respondents to assess SIC practice among adult postoperative patients in public hospitals of Mekelle, Ethiopia 2023/2024 (n=310)**

| Variable | Category | Frequency | Percent |
|---|---|---|---|
| Duration of counseling | <05 minutes | 241 | 77.7 |
| | >05 minutes | 69 | 22.3 |
| | Total | 310 | 100 |
| Qualification of counselor who took surgical informed consent | Senior physician | 68 | 2 |
| | Resident physician | 29 | 9.4 |
| | Intern or GP | 5 | 1.6 |
| | Nurse | 32 | 10.3 |
| | Midwife | 23 | 7.4 |
| | Did not know | 153 | 49.4 |
| | Total | 310 | 100 |
| Schedule of surgery | Elective | 126 | 40.6 |
| | Emergency | 184 | 59.4 |
| | Total | 310 | 100 |
| Type of hospital | Specialized | 194 | 62.6 |
| | General | 116 | 37.4 |
| | Total | 310 | 100 |
| Type of anesthesia | General | 137 | 44.2 |
| | Spinal | 173 | 55.8 |
| | Total | 310 | 100 |
| Timing of counseling for informed consent | The day before date of surgery | 109 | 35.2 |
| | On the day of surgery | 58 | 18.7 |
| | Immediately before surgery | 126 | 40.6 |
| | On the operation table | 17 | 5.5 |
| | Total | 310 | 100 |
| Type of surgery | General surgery | 135 | 43.5 |
| | Orthopedic | 64 | 20.6 |
| | Gynecology/obstetric | 111 | 35.9 |
| | Total | 310 | 100 |

## Components of surgical informed consent received by respondents

Almost all (99%) of the respondents or their family members signed an informed consent form. Most respondents (87.7%) were informed about the indication for the surgery. However, only 8.4% of respondents were informed about the expected surgery duration, and very few respondents (1%) were given a chance to choose their anesthesia. More than half (55.5%) of respondents received information about potential complications during the procedure. *Table 3*

**Table 3: Essential components of surgical informed consent received by respondents to assess SIC practice among adult postoperative patients in public hospitals of Mekelle, Ethiopia 2023/2024 (n=310)**

| Variables | Responses , n, % | | |
|---|---|---|---|
| | Yes | No | Do not remember |
| Respondent/respondent's family was requested for surgical informed consent | 303(97.7) | 5(1.6) | 2(0.6) |
| Respondent/respondent's family signed on an surgical informed consent form | 307(99) | 3(1) | _ |
| Respondent was informed why the surgery will be performed (indication of surgery) | 272(87.8) | 33(10.6) | 5(1.6) |
| Respondent was informed the expected time the surgery will take | 26(8.4) | 273(88.1) | 11(3.5) |
| Respondent was informed about presence/absence of alternative treatment options | 47(15.2) | 254(81.9) | 9(2.9) |
| Respondent was informed about type of anesthesia to be used | 19(6.1) | 275(88.7) | 16(5.2) |
| Respondent was given counseling aids which assist in decision making | 140(45.2) | 163(52.6) | 7(2.2) |
| Respondent was informed about potential complication/s which may arise | 203(65.5) | 100(32.3) | 7(2.2) |
| Respondent was informed about consequences of refusing the proposed surgery | 179(57.7) | 125(40.3) | 6(2) |
| There was a favorable environment to say "No" to the proposed surgery | 58(18.7) | 248(80) | 4(1.3) |
| Respondent was given adequate time for decision to sign on the informed consent form | 164(52.9) | 139(44.8) | 7(2.3) |
| Respondent was given opportunity to choose from anesthesia options | 3(1) | 298(96.1) | 9(2.9) |
| Respondent was given an opportunity to ask question | 127(41) | 179(57.7) | 4(1.3) |

## Receipt of six or more components of surgical informed consent

Based on the total numbers of the components of SIC respondents received before their surgery, out of the total thirteen components 35.8% (CI =95%, 30.6, 41)of the respondents were identified to have received six or more (the minimum recommendation) components of surgical informed consent. *Figure 3*

## Factors associated with receipt of six or more components of surgical informed consent

In bi-variable regression analysis variables like educational level, occupation, sex, type of anesthesia, type of surgery, schedule of surgery, timing of SIC delivery, health care provider who took SIC, hospital type, and counselor time spent on informed consent were significant at P= <0.2, thus they were selected for multi-variable regression analysis. Following multi-variable regression analysis variables like educational level, timing of SIC delivery, Qualification of counselor who took SIC, hospital type, and counselor time spent on informed consent were significant at P<0.05. *Table 4*

**Table 4; Bi-variables and multi-variable analysis to assess factors associated with receipt of six or more components of SIC among adult postoperative patients in public hospitals of Mekelle, Ethiopia 2023/2024 n=310**

| Variable | Category | Received 6 or more components of SIC | | COR (95%CI) | AOR(95% CI) | P-value |
|---|---|---|---|---|---|---|
| | | Yes | No | | | |
| Educational level | able to read and write | 16 | 30 | 1.47(0.69,3.15) | 2.29(0.84,6.2) | 0.105 |
| | Primary(1-8) | 19 | 38 | 1.38(0.67,2.82 | 2.1(0.75,5.9) | 0.157 |
| | Secondary (9-12) | 19 | 41 | 1.28(0.63,2.64) | 2.5(0.68,8.2) | 0.129 |
| | Diploma | 15 | 13 | 3.18(1.33,7.62) | 5.1(0.93,28.15) | 0.06 |
| | Degree and above | 17 | 8 | 5.86(2.25,15.27) | **5.76(1.02,32.6)*** | **0.047*** |
| | Unable to read and write | 25 | 69 | Ref. | Ref. | |
| Duration of counseling | <05 minutes | 61 | 180 | Ref. | Ref. | |
| | >05 minutes | 50 | 19 | 7.765(4.25,14.19) | **65.9(3.33,14.3)*** | **<0.001*** |
| Qualification of counselor who took SIC | Did not know | 48 | 105 | Ref. | Ref. | |
| | Senior physician | 39 | 29 | 2.94(1.63,5.3) | **3.18(1.21,8.38)*** | **0.019*** |
| | Resident physician, Intern or GP | 10 | 24 | 0.91(0.4,2) | 0.445(0.15,1.35) | 0.153 |
| | Nurse | 9 | 23 | 0.856(0.37,1.99) | 1.89(0.59,6.1) | 0.287 |
| | Midwife | 5 | 18 | 0.6(0.21,1.73) | 1.34(0.3,6) | 0.704 |
| Type of hospital | General | 31 | 85 | Ref. | Ref. | |
| | Specialized | 80 | 114 | 1.92(1.16,3.17) | **2.85(1.26,6.46)*** | **0.012*** |
| Timing of counseling for informed consent | Immediately before surgery | 35 | 108 | Ref. | Ref. | |
| | The day before date of surgery | 59 | 50 | 3.64(2.13,6.22) | **3.27(1.5,7.11)*** | **0.003*** |
| | On the day of surgery | 17 | 41 | 1.28(0.65,2.53) | 0.86(0.36,2.1) | 0.751 |
| Occupation | Daily laborer | 4 | 9 | Ref. | Ref. | |
| | Private employee | 6 | 12 | 1.125(0.243,5.2) | 0.51(0.79,3.3) | 0.481 |
| | Farmer | 37 | 77 | 1.08(0.31,3.74) | 1.09(0.21,5.53) | 0.919 |
| | Housewife | 16 | 56 | 0.643(0.175,2.36) | 0.99(0.17,5.6) | 0.988 |
| | Private business | 5 | 18 | 0.625(0.134,2.9) | 0.57(0.85,3.84) | 0.564 |

| | | | | | | |
|---|---|---|---|---|---|---|
| | Government employee | 29 | 16 | 4.08(1.08,15.37) | 1.59(0.24,10.5) | 0.629 |
| | Other | 14 | 11 | 2.86(0.69,11.82) | 2.34(0.43,12.8) | 0.326 |
| Sex | Female | 48 | 102 | Ref. | Ref. | |
| | Male | 63 | 97 | 1.38(0.8,2.2) | 0.92(0.37,2.3) | 0.86 |
| Schedule of surgery | Elective | 51 | 75 | 1.4(0.88,2.25) | 1.01(0.49,2.07) | 0.977 |
| | Emergency | 60 | 124 | Ref. | Ref. | |
| Type of surgery | General surgery | 53 | 82 | Ref. | Ref. | |
| | Orthopedic | 30 | 34 | 1.37(0.75,2.5) | 1.28(0.55,2.98) | 0.565 |
| | Gynecology/obstetric | 28 | 83 | 0.52(0.3,0.9) | 0.76(0.24,2.4) | 0.641 |
| Type of anesthesia | General | 59 | 78 | 1.76(1.1,2.81) | 1.07(0.51,2.24) | 0.854 |
| | Spinal | 52 | 121 | Ref. | Ref. | |

***= Statistically significant with p- value < 0.2** **1=Reference group of the variable**

****=Statistically significant with p- value <0.05**

# Discussion

The receipt of six or more (the minimum recommendation) components of SIC in this study is (35.8%) with (CI 95%, 30.6, 41), which is slightly higher than the study conducted at Hawassa (26.5%) (8). This difference might be attributable to the study area covered, which exposes patients to various health professionals and different working environments. This is because this study included all public hospitals and post-op wards, whereas the latter study was exclusively conducted in the gynecology/obstetrics department of a specific hospital. On the other hand, it is lower than the study conducted in Addis Ababa (44.9%) (9). This disparity is likely due to decreased staff morale in helping patients in the study area, possibly stemming from a lack of incentives, service delivery arrangements, resource capacities and increased workload (5).

A multi-variable regression analysis identified several factors associated with receipt of six or more components of SIC. Participants with a degree or higher educational level were 5.7 times more likely to receive 6 or more components than those who were unable to read or write (AOR=5.76, 95%CI=1.02, 32.6, P=0.047)**.** These findings are in line with a study conducted in Iran, and Addis Ababa (AOR=2.4, 95%CI=1.81, 11.32) (9, 11). This difference might be attributable to the fact that educated participants tend to ask more questions. This, in turn, led them to receive more information about the proposed surgery.

Participants counseled for more than 5 minutes were almost seven times more likely to receive the 6 or more components of SIC than those counseled for less than 5 minutes (AOR= 6.9, 95% CI =3.33,14.3,). This is comparable to the study conducted in Addis Ababa which is two times (AOR=1.8, 95%CI=1.79, 3.69) (9). These findings suggest that when health professionals devote

more time to patients, the delivery of key SIC components significantly increases as evidenced by a study conducted in south eastern Ethiopia (5)**.**

Participants counseled one day before surgery were three times more likely to receive the six or more components of SIC compared to those counseled immediately before surgery (AOR=3.27, 95%CI=1.5, 7.11, P=0.003). This finding is in line with a study conducted in Hawassa as 80% of patients who received SIC immediately before surgery were less likely to receive the six or more components of SIC (OR: 0.02; 95% CI: 0.01–0.10) (8). This shows patients counseled earlier before surgery has adequate time to discuss their surgery, thus they are not rushed to undergo surgery with limited information.

Participants who underwent surgery at a specialized hospital were 2.8 times more likely to receive six or more components of SIC than those who went to general hospitals (AOR = 2.85, 95% CI = 1.26, 6.46 P= 0.012). This may be due to specialized hospital often have greater staffing and resources, including students who can provide additional information about the surgery.

Participants who were counseled by a senior physician are 3 times more likely to receive six or more components of SIC than those who didn’t know who counseled them (AOR = 3.185, 95% CI = 1.21, 8.38, P = 0.019). This might be senior physicians tend to offer more comprehensive explanations about the procedure and hold primary responsibility for their patients' overall health (6).

## Limitations of the study

Recall bias was a concern as patients were interviewed after surgery. As it was cross-sectional study, doesn’t show the cause and effect relationship. Finally, this study was conducted only in Mekelle city public hospitals, Tigray, the generalizability of its results to other zones of Tigray may be limited.

## Conclusion

This study showed that majority of participants who underwent both elective and emergency surgery did not receive comprehensive information during the SIC process in the study hospitals. The absence of thorough and standardized pre-operative counseling for surgical procedures undermines, the quality of care patients receive and ability of healthcare facilities to meet patient expectations and information needs. Educational level, timing of SIC delivery, qualification of counselor who took SIC, hospital type, and counselor time spent on informed consent had significant effect on receipt of six or more components of SIC.

To improve SIC delivery it is suggested that health professionals; create rapport with patient, spend more time during counseling, and deliver it early.

## Availability of data and materials

All data are in the manuscript and/or supporting information file.

## Competing interest

The authors declare that they have no competing interests

## Funding

No funding’s

## Author’s contribution

FA – developed the proposal, designed and participated in data collection, conducted the data analysis and interpretation, developed the first draft, revised subsequent drafts, and wrote the manuscript. HK- advised on the conception of the research proposal, data analysis, interpretation, reviewed and commented on successive drafts. DB- advised on the development of the research proposal, data analysis, interpretation, reviewed and commented on successive drafts. BT – participated in the data collection, reviewing and editing the original draft. FM – participated in the data analysis, and data curation. MA participated in the data analysis, and data curation. All authors reviewed and approved the final draft of the manuscript.

## Authors information

FG (MSc. in adult health Nursing) lecturer in Aksum University; HK (PhD) Associate professor in Mekelle University; DB (MSc. in adult health Nursing) Assistant Professor in Mekelle University; BT (MSc. in adult health Nursing) lecturer in Adigrat University; FM (MSc. in adult health Nursing) lecturer in Gambella teachers’ education and health science college. MA (MSc. in adult health Nursing) lecturer in Adigrat University.

## Abbreviations

AOR – Adjusted Odds Ratio; CRD- Crude; Odds Ratio; E.C- Ethiopian calendar; EMA- Ethiopian Medical Association; G.C- Gregorian calendar ; GH- General Hospital; IRB- Institutional Review Board; MCC- Motivated Competent Compassionate; MGH- Mekelle General Hospital; OR – Odds Ratio; SIC- Surgical Informed Consent; SPSS: Statically Package Software for Social Science; UK – United Kingdom.

## Acknowledgement

First of all, I would like to express my sincere gratitude to the study participants for their willingness to participate in the study, and to my data collectors and supervisors for their facilitation, organization and collection of the data throughout the study period.

Finally, I extend my sincere thanks to Aksum University for providing me with the opportunity to expand my knowledge and refine my skills. I am also grateful to Mekelle University, College of Health Sciences, School of Nursing for their support in completing my thesis report.

# Reference


1. Farmer L, Lundy A. Informed consent: Ethical and legal considerations for advanced practice nurses. The Journal for Nurse Practitioners. 2017;13(2):124-30.
2. Fields LsI. The Royal College of Surgeons of England. Consent: supported decision making. A guide to good practice. London: The Royal College of Surgeons;. 2016.
3. Leclercq WK, Keulers BJ, Scheltinga MR, Spauwen PH, van der Wilt G-J. A review of surgical informed consent: past, present, and future. A quest to help patients make better decisions. World journal of surgery. 2010;34(7):1406-15.
4. Ochieng J, Buwembo W, Munabi I, Ibingira C, Kiryowa H, Nzarubara G, et al. Informed consent in clinical practice: patients' experiences and perspectives following surgery. BMC research notes. 2015;8(1):1-6.
5. Negash W, Assefa N, Baraki N, Wilfong T. Practice and factors associated with informed consenting process for major surgical procedures among health-care workers, south eastern Ethiopia. International Journal of General Medicine. 2021:7807-17.
6. Rajesh D, Singh A, Chopra M, Singh G, Venkteshan M, Bhardwaj A, et al. Patients awareness, attitude, understanding and perceptions towards legal nature of informed consent. J Indian Acad Forensic Med. 2013;35(1):0971-3.
7. Ogundiran TO, Adebamowo CA. Surgeons' opinions and practice of informed consent in Nigeria. Journal of medical ethics. 2010;36(12):741-5.
8. Teshome M, Wolde Z, Gedefaw A, Tariku M, Asefa A. Surgical informed consent in obstetric and gynecologic surgeries: experience from a comprehensive teaching hospital in southern Ethiopia. BMC medical ethics. 2018;19(1):1-9.
9. Kebede E, Tasew T. Surgical Informed Consent in Clinical Practice: Patients' Perspective Undergoing Cesarean Section at Three Teaching Hospitals in Addis Ababa, Ethiopia. Ethiopian Journal of Health Sciences. 2023;33(4).
10. Gebregziabher M, Amdeselassie F, Esayas R, Abebe Z, Silvia H, Teklehaimanot AA, et al. Geographical distribution of the health crisis of war in the Tigray region of Ethiopia. BMJ global health. 2022;7(4):e008475.
11. Sheikhtaheri A, Farzandipour M. Factors associated with quality of informed consent in patients admitted for surgery: an Iranian study. AJOB Primary Research. 2010;1(4):9-16.
12. Convie L, Carson E, McCusker D, McCain R, McKinley N, Campbell W, et al. The patient and clinician experience of informed consent for surgery: a systematic review of the qualitative evidence. BMC Medical Ethics. 2020;21(1):1-17.
13. MOH E. Federal Democratic Republic of Ethiopia Ministry of Health Health Sector Development Program IV October 2010 Contents. October 2010. 2014.
14. Cawich SO, Barnett AT, Crandon IW, Drew SD, Gordon-Strachan G. From the patient's perspective: is there a need to improve the quality of informed consent for surgery in training hospitals? The Permanente Journal. 2013;17(4):22.

# Response to Reviewers

1. I have summarized parts of the methods and materials in one paragraph. i.e.; study area, period, population and design etc…
2. I have also removed unnecessary parts from methods and materials such as dissemination of results.
3. I have amended the manuscript based on Plos one templates ad guidelines.
4. I have given each figure its own caption.
5. I have changed data availability as advised.
