# Supplementary material for: Surgical informed consent practice and associated factors among adult postoperative patients in public hospitals of Mekelle, Tigray, Ethiopia 2023/2024. Cross sectional
Source: PLoS One. 2025 Feb 3;20(2):e0310920. doi: 10.1371/journal.pone.0310920 (PMC11790128; doi:10.1371/journal.pone.0310920)
Supplement: Attachment — Submitted filename: Response to Reviewers.docx [file pone.0310920.s002.docx]

**Response to Reviewers**

1. I have summarized parts of the methods and materials in one paragraph. i.e.; study area, period, population and design etc…
2. I have also removed unnecessary parts from methods and materials such as dissemination	of results.
3. I have amended the manuscript based on Plos one templates ad guidelines.
4. I have given each figure its own caption.
5. I have changed data availability as advised.
