# Supplementary material for: Surgical informed consent practice and associated factors among adult postoperative patients in public hospitals of Mekelle, Tigray, Ethiopia 2023/2024. Cross sectional
Source: PLoS One. 2025 Feb 3;20(2):e0310920. doi: 10.1371/journal.pone.0310920 (PMC11790128; doi:10.1371/journal.pone.0310920)
Supplement: Attachment — Submitted filename: Response to Reviewers.docx [file pone.0310920.s005.docx]

**Response to Reviewers**

1. I have included the key words.
2. I have removed the repeated parenthesis.
3. I have revised the write up of the study area, population, sample size and procedure.
4. I have written the exclusion and inclusion criteria, structurally and academically.
5. I have summarized study variable into one paragraph.
6. I have summarized study tool into one paragraph.
7. I have removed the operational definition.
8. I have written the reliability, normality and validity tools.
9. I used 5% of my sample for pretest by referring to similar studies.
10. I have amended word on the methodology part.
11. I have determined my data normality using SPSS software (by conducting Q_Q plot and Kolmogorov-Smirnov test) .
12. I have determined to retain variables from bivariate to multivariate regression using P-value which is <0.2.
13. I have revised the discussion part.
14. I have used recent and available references.
15. I have checked the grammar and flow of my write up using AI.
