# Supplementary material for: Surgical informed consent practice and associated factors among adult postoperative patients in public hospitals of Mekelle, Tigray, Ethiopia 2023/2024. Cross sectional
Source: PLoS One. 2025 Feb 3;20(2):e0310920. doi: 10.1371/journal.pone.0310920 (PMC11790128; doi:10.1371/journal.pone.0310920)
Supplement: Attachment — Submitted filename: Reviewer comments .pdf [file pone.0310920.s003.pdf]

Review comments

Title: Surgical informed consent practice and associated factors among adult postoperative patients in public hospitals of Mekelle, North Ethiopia 2023/2024. Cross sectional

Comments

Good choice for a paper title, it's essential to discuss the details, risks, and potential side effects of the procedure with the patient, as it is an ethical and moral issues. But it needs some modification, as previous studies were limited to local studies only and did not address researches and results at the global level. The following is step by step comments.

In paragraph no 26

Add the methods of data collection tool(s) and techniques to this section

In paragraph no 36

Add the value of significance

In paragraph no 56

The statement is not aligned with the reference mentioned, or add ref for the Italy study.

In paragraph 85 - 86

Revise the statement in this paragraph

In paragraph no 110

The number of hospitals to be studied must be specified.

In paragraph no 150

Write about the reliability and validity of your tool

In paragraph no 200

Match it with data collection tool   in paragraph no 150

Comments: The study relied only on limited previous studies at the Ethiopian level only and one Italian. The author must expand a little on: international, national, regional studies and finally on the local studies.

Also, the recommendations are simple and modest compared to the results of this study. More recommendations related to finding are needed.

Good luck
